# Electrospray Ionization Mass Spectrometric Analysis of Highly Reactive Glycosyl Halides

**DOI:** 10.3390/molecules17078351

**Published:** 2012-07-10

**Authors:** Attila Bokros, Annamária Bánfi, Zoltán Kupihár, Zoltán Kele, Tünde Zita Illyés, János Szolomájer, Lajos Kovács

**Affiliations:** 1 Department of Medicinal Chemistry, Faculty of Medicine, University of Szeged, Dóm tér 8, H-6720 Szeged, Hungary; Email: bokros.attila@med.u-szeged.hu (A.B.); banfi.annamaria@med.u-szeged.hu (A.B.); kele.zoltan@med.u-szeged.hu (Z.K.); szolomajer.janos@med.u-szeged.hu (J.S.); 2 Department of Organic Chemistry, Faculty of Science and Technology, University of Debrecen, Egyetem tér 1, H-4010 Debrecen, Hungary; Email: illyes.tunde@science.unideb.hu

**Keywords:** glycosyl halides, electrospray mass spectrometry, nanoelectrospray, lithium salts

## Abstract

Highly reactive glycosyl chlorides and bromides have been analysed by a routine mass spectrometric method using electrospray ionization and lithium salt adduct-forming agents in anhydrous acetonitrile solution, providing salient lithiated molecular ions [M+Li]^+^, [2M+Li]^+^*etc.* The role of other adduct-forming salts has also been evaluated. The lithium salt method is useful for accurate mass determination of these highly sensitive compounds.

## 1. Introduction

Carbohydrates are the most abundant organic compounds on Earth. Their multifaceted role is underlined, among others, by the extensive and varied glycosylation pattern they display in a number of conjugates (glycoproteins, proteoglycans, glycolipids, glycoproteolipids *etc.*). Clearly, glycosylation is one of the most important reactions of carbohydrate derivatives. For the chemical glycosylation different active (or activatable) glycosyl donors are available (glycosyl halides, trichloroacetimidates, thioglycosides, orthoesters, glycals, *n*-pentenyl glycosides *etc.*) [1,2]. Historically, glycosyl halides emerged as the first efficient glycosylating donors in the Koenigs-Knorr method as early as 1901, the glycosylation taking place in the presence of a heavy metal salt [3]. Although the role of these glycosyl halides is somewhat restricted in contemporary glycosylation procedures, glycosyl fluorides and glycosyl iodides are still often used. Comparatively, glycosyl fluorides are the most stable glycosyl halides [4,5], the chlorides and bromides are endowed with increased reactivity and decreased stability against nucleophiles, however, they can be isolated and stored for a limited time under anhydrous conditions and low temperatures [1,2]. The glycosyl iodides are very reactive, mostly very unstable and are used *in situ* in glycosylation reactions [1,6].

The analytical characterization of glycosyl halides is very limited by their stability. The most often used glycosyl chlorides and bromides are sometimes characterized by NMR and IR [7,8,9]. The elemental analysis and mass spectrometric characterization of glycosyl halides is greatly hampered by their moisture sensitivity and in the routinely used electrospray mass spectrometry cocktails containing alcohols, water and acids they decompose rapidly, as well as under the high-temperature conditions of harsher ionization techniques (e.g., electron impact).

Owing to these difficulties the mass spectrometric characterization of glycosyl halides is very scarce in the literature and is limited to the tandem electrospray mass spectrometric investigation of the relatively stable glycosyl fluorides published in Russian [10,11].

Our previous experience in the nanoelectrospray mass spectrometric investigation of similarly acid- and nucleophile-sensitive phosphoramidites employing lithium chloride adduct ions [12] spurred us to consider the application of our method to selected glycosyl chlorides and bromides. In a later development we have been able to extend the scope of our lithium chloride method to the high resolution MS characterization of phosphoramidites [13], thus the accurate mass determination allowed us to confirm the elemental composition of analytes. In the present paper we would like to disclose our results on the ESI-MS characterization and accurate mass measurement of selected glycosyl chloride and bromide analytes.

## 2. Results and Discussion

### 2.1. Sample Preparation

**Figure 1:**
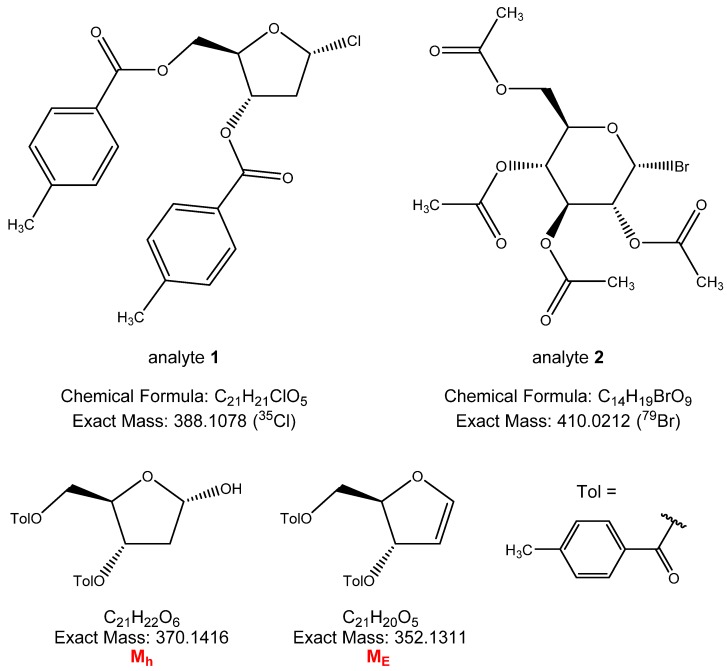
The structures of the analyte glycosyl halides **1** and **2** and the decomposition products of glycosyl chloride **1**.

For the mass spectrometric analysis two model compounds, a glycosyl chloride and a glycosyl bromide were chosen: 2-deoxy-3,5-di-*O*-*p*-toluoyl-α-D-*erythro*-pentofuranosyl chloride (analyte **1**) and 2,3,4,6-tetra-*O*-acetyl-α-D-glucopyranosyl bromide (analyte **2**). Their structures can be seen in Figure 1*.* 2-Deoxy-3,5-di-*O*-*p*-toluoyl-α-D-*erythro*-pentofuranosyl chloride [14] is often used in the synthesis of 2'-deoxynucleosides while 2,3,4,6-tetra-*O*-acetyl-α-D-glucopyranosyl bromide [15] is the most common glycosyl halide.

Based on our earlier experience [12], anhydrous acetonitrile was selected as a solvent because it is frequently used in carbohydrate chemistry, compatible with the ESI-MS instruments, the protected glycosyl halides are highly soluble in it, and it can dissolve the required amount of adduct-forming lithium salt.

The samples were prepared freshly, right before the analysis to avoid the potential decomposition due to the moisture traces in the solvent and the hygroscopic property of the acetonitrile. When the sample was prepared 30 minutes before the analyses, usually glycosyl halides could not be detected.

A relatively high concentration of the analyte in the sample can cause problems in the formation of adduct-ions, poor resolution and peak accuracy in the spectrum, therefore we have tried to use a very low analyte concentration at the beginning. Unfortunately, even using anhydrous acetonitrile, the small water content was enough to decompose the whole amount of analyte when a relatively high amount of solvent was used, therefore increasing the concentration of the analyte was necessary to get reasonable spectra.

### 2.2. Measurement of Glycosyl Chloride and Bromide Samples

The sample introduction to the ion source was tried in two ways: first we used gold-coated fused borosilica capillary to minimize the amount of the used solvent and contaminations but then for the accurate mass determination we used the normal ionspray with direct injection to get more constant signals parallel with the built-in lockmass standard signal produced by the secondary ion spray. We used anhydrous acetonitrile as eluent in the pump and applied the possibly smallest dead volume between the injector and the ion source to minimize the potential decomposition of the analyte. We did not find significant differences between the two sample introduction methods. In both cases the spectra contained the desired molecule-adduct-ion peaks with relatively high intensities.

**Figure 2:**
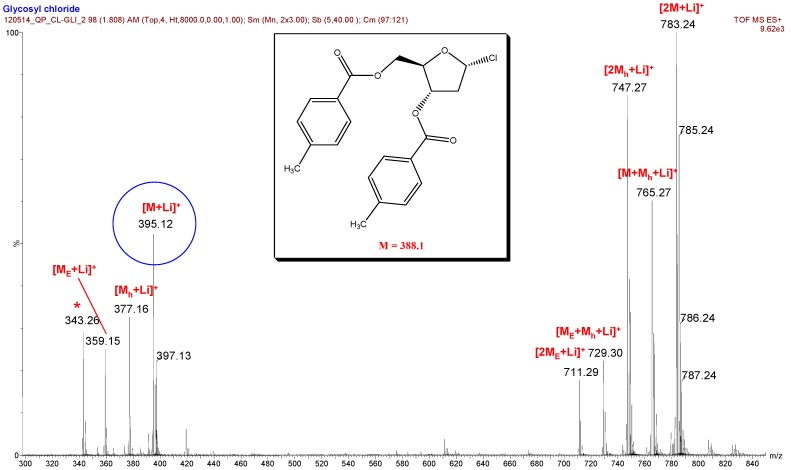
Mass spectrum of glycosyl chloride analyte **1** (* indicates an unidentified impurity).

**Figure 3:**
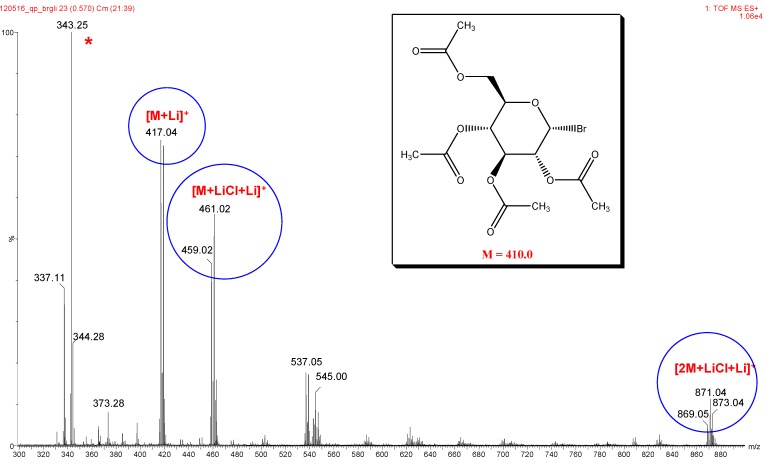
Mass spectrum of analyte glycosyl bromide **2** (* indicates an unidentified impurity).

Beside the desired [M+Li]^+^, [2M+Li]^+^ or [M+LiCl+Li]^+^ peaks, the glycosyl halide hydrolysis peaks ([M_h_+Li]^+^), hydrogen halide elimination peaks [M_E_+Li]^+^ (cf. Figure 1), combined associated peaks ([M+M_h_+Li]^+^, [M_E_+M_h_+Li]^+^) can also be seen in the spectra of analyte **1** in Figure 2. Analyte **2** did not show similar decomposition pattern (Figure 3).

The formation of hydrolyzed by-product is likely attributed to the appearance of a trace amount of water present in anhydrous acetonitrile that could not be excluded in spite of careful drying. The measured *m/z* values and the isotopic distributions of the peak serials clearly support the assumed composition of the ions.

Based on our earlier experience with sensitive phosphoramidites [12,13], we started our experiments with lithium chloride as an adduct-forming agent. However, we also examined the cases without adduct-forming ions, with formic acid (used in routine ESI cocktails) and other salts (LiF, LiBr, LiI, LiNO_3_, LiClO_4_, NaCl, NaNO_3_, NaClO_4_, KCl, KNO_3_, KClO_4_, NH_4_Cl, NH_4_NO_3_, NH_4_HCO_3_, NH_4_OOCH, NH_4_OOCCH_3_) in order to investigate the formation of positive and negative ions and the influence of anions.

In the absence or presence of formic acid (without adduct-forming salts) very weak signals have been obtained compared to the spectra recorded with application of salts (Supplementary Information, spectra 1 and 2). When comparing lithium, sodium, potassium and ammonium salts (chloride, nitrate, perchlorate), the lithium salts gave the most prominent peaks in the spectra (Supplementary Information, spectra 3–11). The ammonium salts (NH_4_Cl, NH_4_NO_3_, NH_4_HCO_3_, NH_4_OOCH, NH_4_OOCCH_3_), popular adduct-forming agents due to their volatility, gave very poor signal intensity of ammonium adduct ions, usually the traces of sodium and potassium peaks were more abundant (Supplementary Information, spectra 12–16). Unidentified peaks designated by asterisks * and ** have been used as a kind of internal standards to compare the signal intensities of lithium, sodium, potassium adduct ions. In respect of lithium salts (LiF, LiBr, LiI, LiNO_3_, LiClO_4_, Supplementary Information, spectra 3, 6, 9, 17, 18) there was no appreciable difference among the spectra therefore eventual anion exchange [16,17] with the reactive glycosyl halide **2** can be excluded. It is conceivable that during the sample preparation in solution phase and in the ESI ion source the very short contact times did not allow anion exchange reaction to take place. However, if a special application requires the negative counterion can be replaced by inert NO_3_^−^ or ClO_4_^−^ ions.

We have also tried the negative ionization mode, but unfortunately we were not able to identify any peaks associated to the adduct ions of the sample, only the cluster ion peak series of the adduct-forming salts (Supplementary Information, spectra 19–23).

The spectra contained peaks with an extra adduct (120 Da neutral mass) the tandem mass spectra of which revealed their identity as a lithium trifluoroacetate adduct. This impurity originates from routine usage of our mass spectrometer employing trifluoroacetic acid that cannot be efficiently removed even after successive washing from the ion source capillary (Supplementary Information, spectra 26, 27).

### 2.3. Accurate Mass Determination

**Figure 4:**
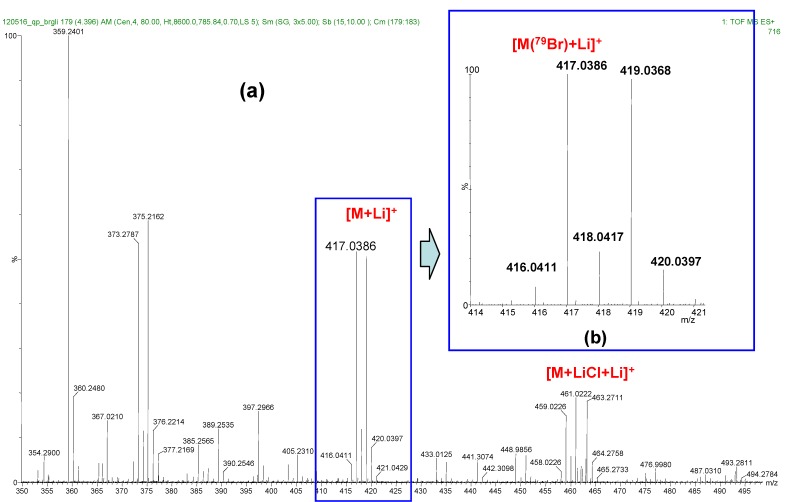
(**a**) A calibrated spectrum of analyte **2** and (**b**) the zoomed [M+Li]^+^ peaks for accurate mass determination.

For the accurate mass determination we have used both the glycosyl chloride **1** and bromide **2** analytes and the built-in lockmass method applying glufibrinopeptide B standard (Supplementary Information, spectrum 24, Table 25). The calibrated spectrum and the zoomed [M+Li]^+^ intense doubled signals (containing ^79^Br and ^81^Br isotopes) can be seen in Figure 4.

The measured and calculated values for both highly intense peaks in Table 1 show that the mass differences are lower than 5 ppm, which is acceptable to confirm the elemental composition of the desired analyte.

**Table 1:** The high-resolution measured and calculated masses of [M+Li]^+^ peaks of analytes **1** and **2**.

Analyte	Formula	Calculated *m/z*	Measured *m/z*	Mass difference (ppm)
1	C_21_H_21_^35^Cl^7^LiO_5_^+^	395.1238	395.1256	4.6
2	C_14_H_19_^79^Br^7^LiO_9_^+^	417.0372	417.0386	3.4

## 3. Experimental

### 3.1. General

Analyte **1**: 2-deoxy-3,5-di-*O*-*p*-toluoyl-α-D-*erythro*-pentofuranosyl chloride [14] and analyte **2**: 2,3,4,6-tetra-*O*-acetyl-α-D-glucopyranosyl bromide [15] have been prepared according to literature methods. All mass spectrometric measurements were performed on a Waters Q-TOF Premier spectrometer (Waters, Milford, MA, USA) equipped with a built-in nanoelectrospray ion source [12]. A high voltage of ca. 1,000 V was used in the ion source. The instrument was scanned in the normal MS mode over the mass range 50–990 with a scan time of 2 s. Nanospray voltage: 1 kV, spray voltage (in case of direct infusion): 3.3 kV. Injection speed: 200 μL/min. Cone voltage: 32 V. Lockmass standard: glu-fibrinopeptide B. All the theoretical masses were calculated by the MassLynx software.

Anhydrous acetonitrile solvent was purchased from Aldrich and stored over 4 Å molecular sieves [18]. The salts used were dried according to standard methods [18]. Saturated salt solutions were prepared using anhydrous acetonitrile as solvent. The solution was centrifuged and the supernatant was diluted with the sample solution in a volume ratio of 1:5. A few microliters of these solutions were then loaded into a pulled, gold-coated borosilicate capillary and analyzed using both the positive and negative ion modes [12,13].

## 4. Conclusions

We have demonstrated that a routine mass spectrometric method using electrospray ionization and lithium salt adduct-forming agents in anhydrous acetonitrile solution is capable of detecting the lithiated molecular ions of glycosyl chlorides and bromides. The comparison of lithium, sodium, potassium and ammonium salts (chloride, nitrate, perchlorate) demonstrated that lithium salts gave the most prominent peaks. Furthermore, the method is useful for accurate mass determination of these highly sensitive compounds. Although the mass spectrometric behaviour of the above substances seems to be rather limited, our experience with phosphoramidites, sensitive towards nucleophiles and acids [12,13], supports the incentive that lithium-ion mediated electrospray mass spectrometry is a suitable tool for the characterization of highly sensitive substances under anhydrous conditions. 

## Supplementary Materials

Supplementary materials can be accessed at: http://www.mdpi.com/1420-3049/17/7/8351/s1.
