# Supplementary material for: Electrospray Ionization Mass Spectrometric Analysis of Highly Reactive Glycosyl Halides
Source: Molecules. 2012 Jul 10;17(7):8351–8. doi: 10.3390/molecules17078351 (PMC6268470; doi:10.3390/molecules17078351)

Molecules **2012**, *17*, 8351-8358; doi:10.3390/molecules17078351

OPEN ACCESS

molecules

ISSN 1420-3049

www.mdpi.com/journal/molecules

Communication

Electrospray Ionization Mass Spectrometric Analysis of Highly Reactive Glycosyl Halides

Attila Bokros [1], Annamária Bánfi [1], Zoltán Kupihár [1,*], Zoltán Kele [1], Tünde Zita Illyés [2], János Szolomájer [1] and Lajos Kovács [1,*]

[1] Department of Medicinal Chemistry, Faculty of Medicine, University of Szeged, Dóm tér 8, H-6720 Szeged, Hungary; E-Mails: bokros.attila@med.u-szeged.hu (A.B.); banfi.annamaria@med.u-szeged.hu (A.B.); kele.zoltan@med.u-szeged.hu (Z.K.); szolomajer.janos@med.u-szeged.hu (J.S.)

[2] Department of Organic Chemistry, Faculty of Science and Technology, University of Debrecen, Egyetem tér 1, H-4010 Debrecen, Hungary; E-Mail: illyes.tunde@science.unideb.hu

* Authors to whom correspondence should be addressed; E-Mails: kupihar.zoltan@med.u-szeged.hu (Z.K.); kovacs.lajos@med.u-szeged.hu (L.K.); Tel.: +36-62-545-145; Fax: +36-62-545-971.

Received: 18 May 2012; in revised form: 21 June 2012 / Accepted: 25 June 2012 / Published: 10 July 2012

Supplementary Information

- Unidentified peaks designated by asterisks * and ** have been used as a kind of internal standards to compare the signal intensities of lithium, sodium, potassium and ammonium adduct ions;
- Identified peaks have labelled in red;
- The desired molecular ion adducts $[M+cation]^+$ are shown in blue circles ◯.

Spectrum 1. Analyte **2** in acetonitrile without any adduct forming agent.

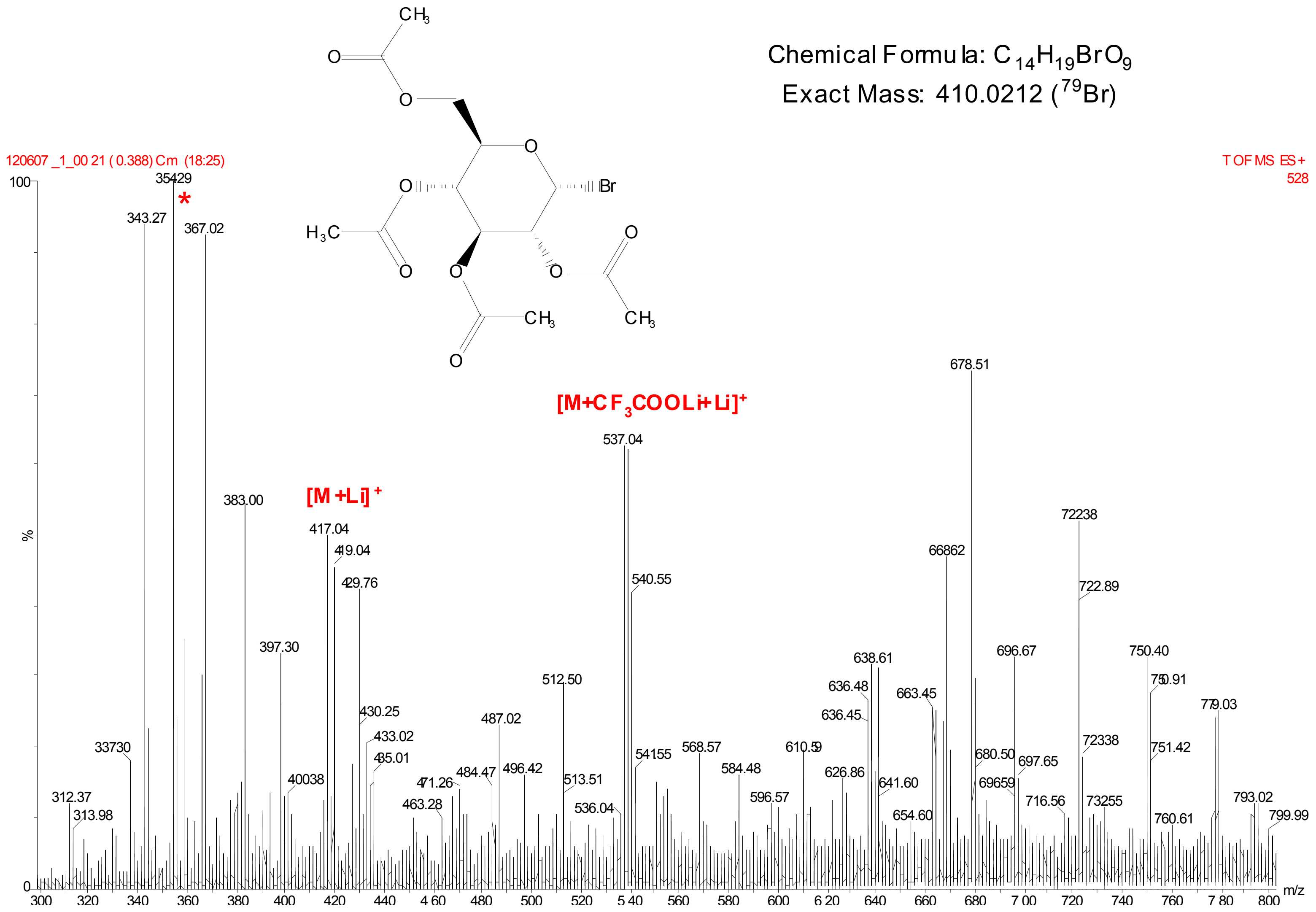

Spectrum 2. Analyte **2** in acetonitrile with 2% formic acid.

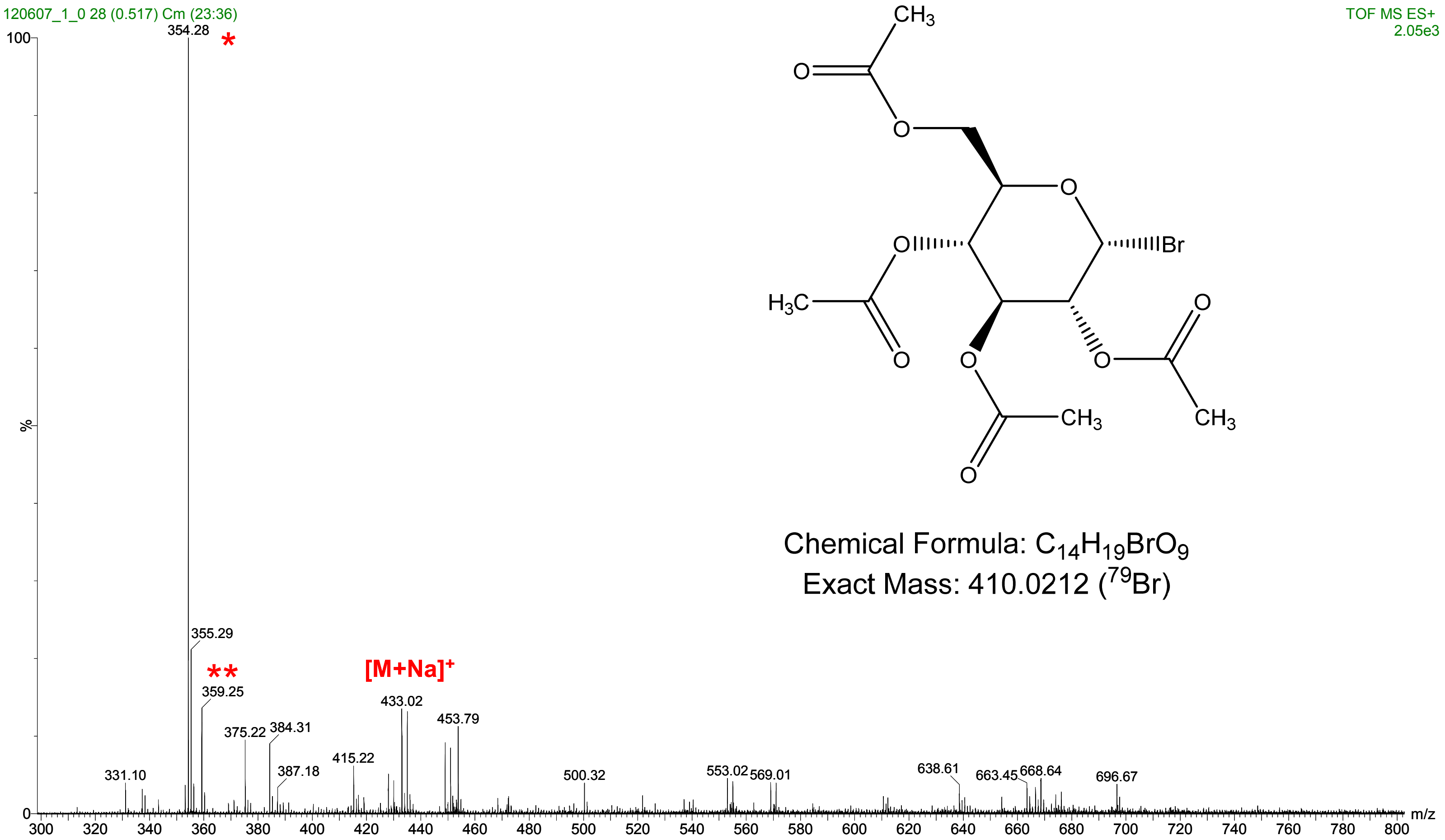

Spectrum 3. Analyte **2** in acetonitrile with LiCl.

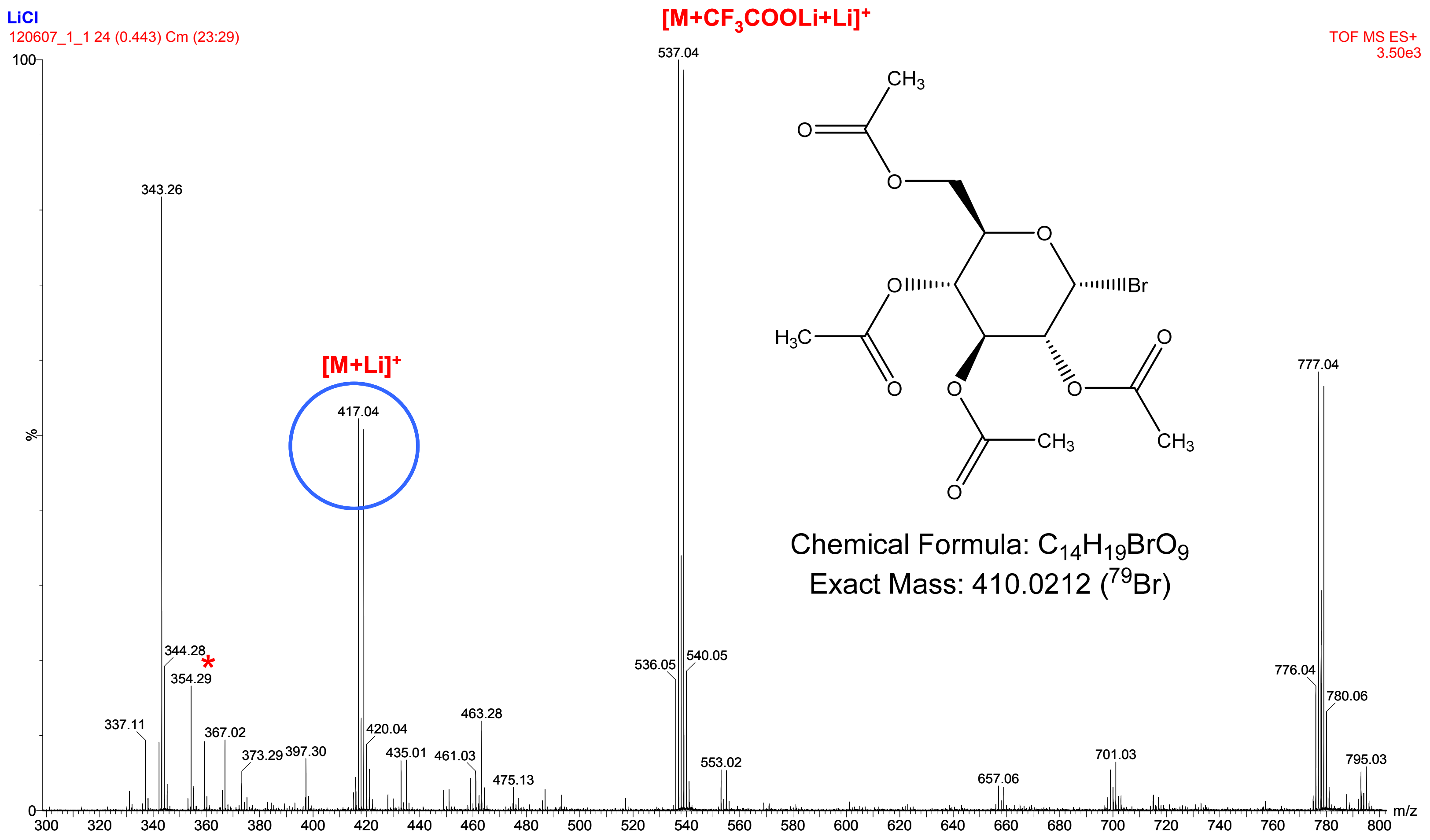

Spectrum 4. Analyte **2** in acetonitrile with NaCl.

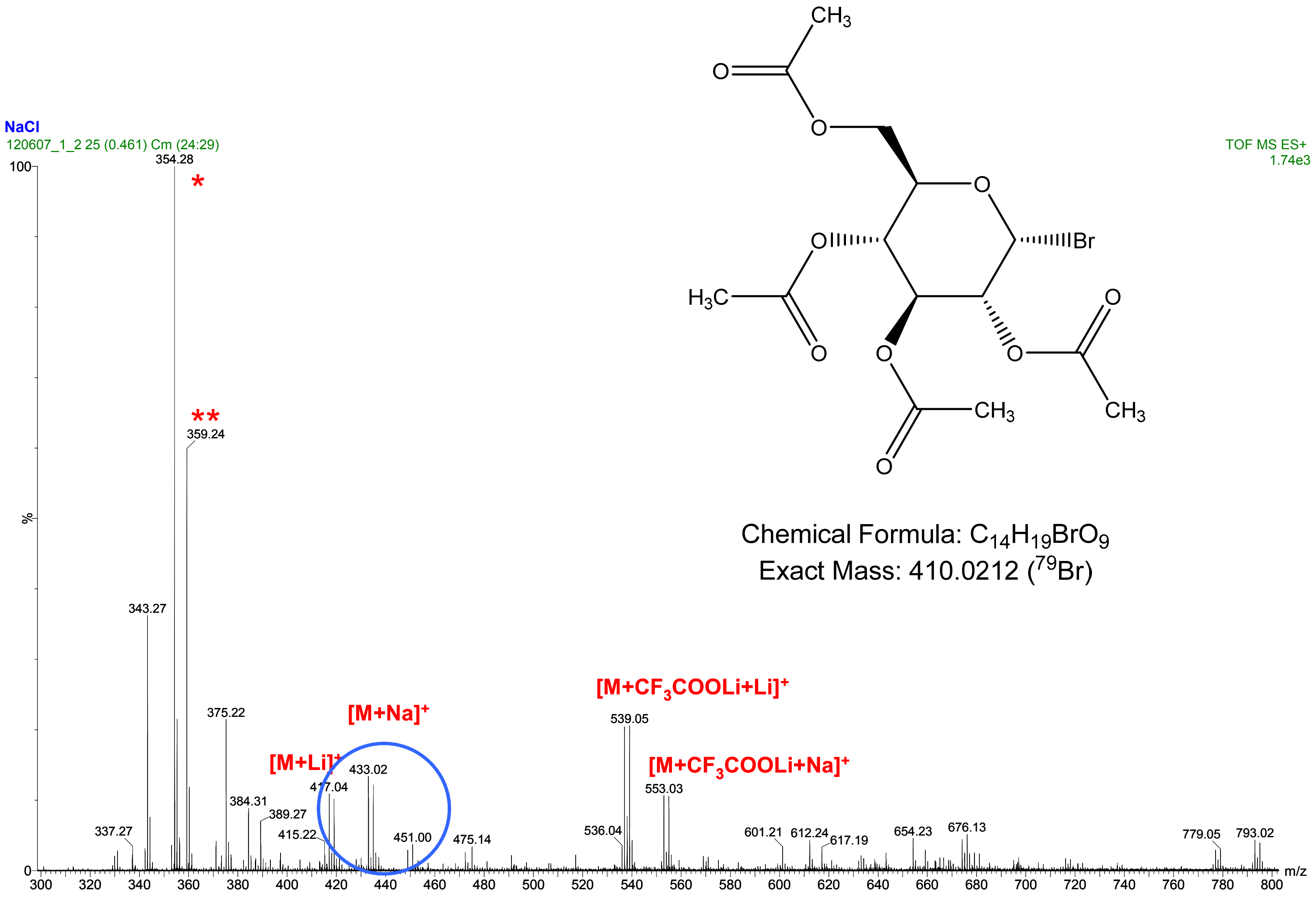

Chemical Formula: $C_{14}H_{19}BrO_9$

Exact Mass: 410.0212 (^{79}Br)

Spectrum 5. Analyte **2** in acetonitrile with KCl.

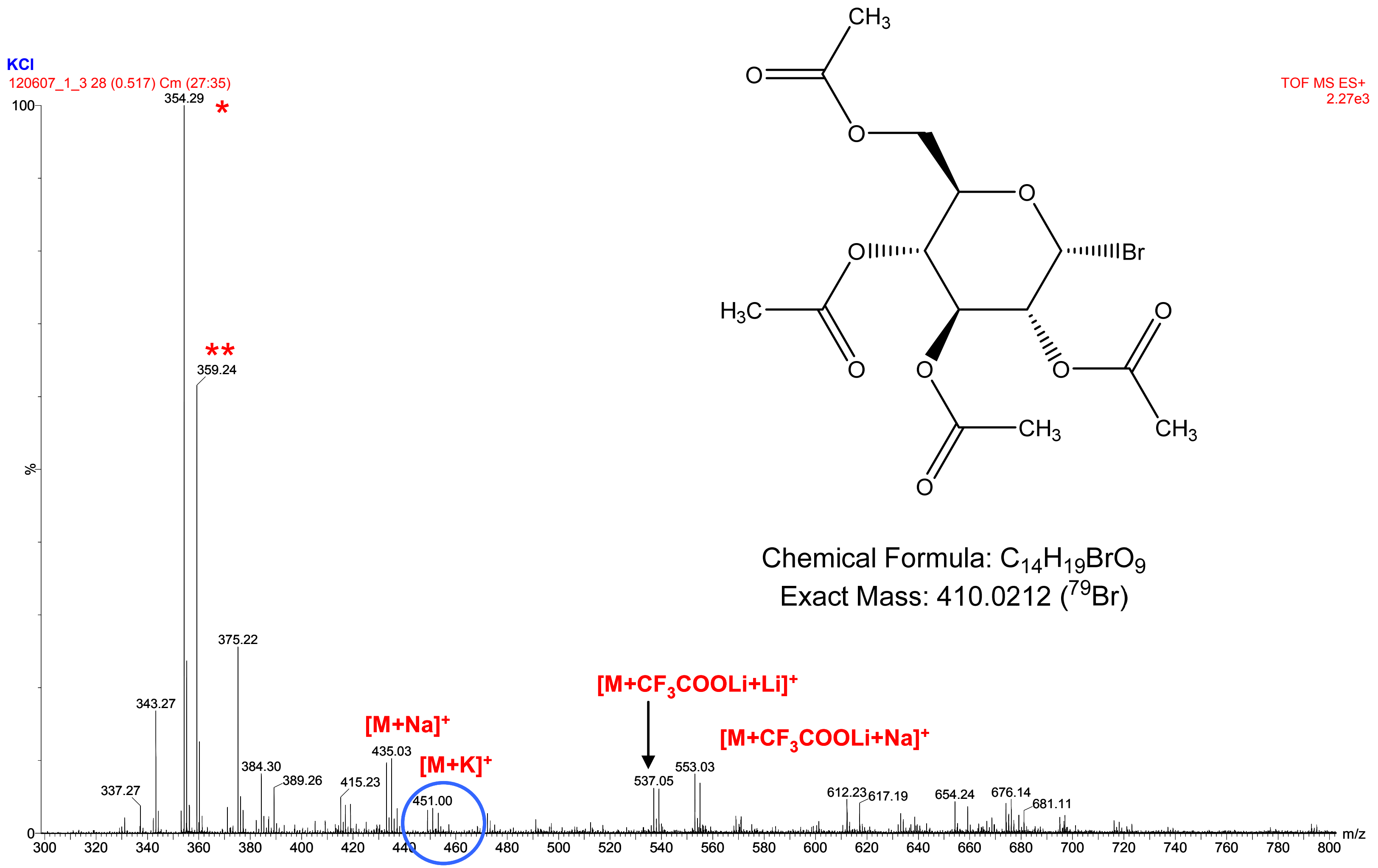

Spectrum 6. Analyte **2** in acetonitrile with $LiNO_3$.

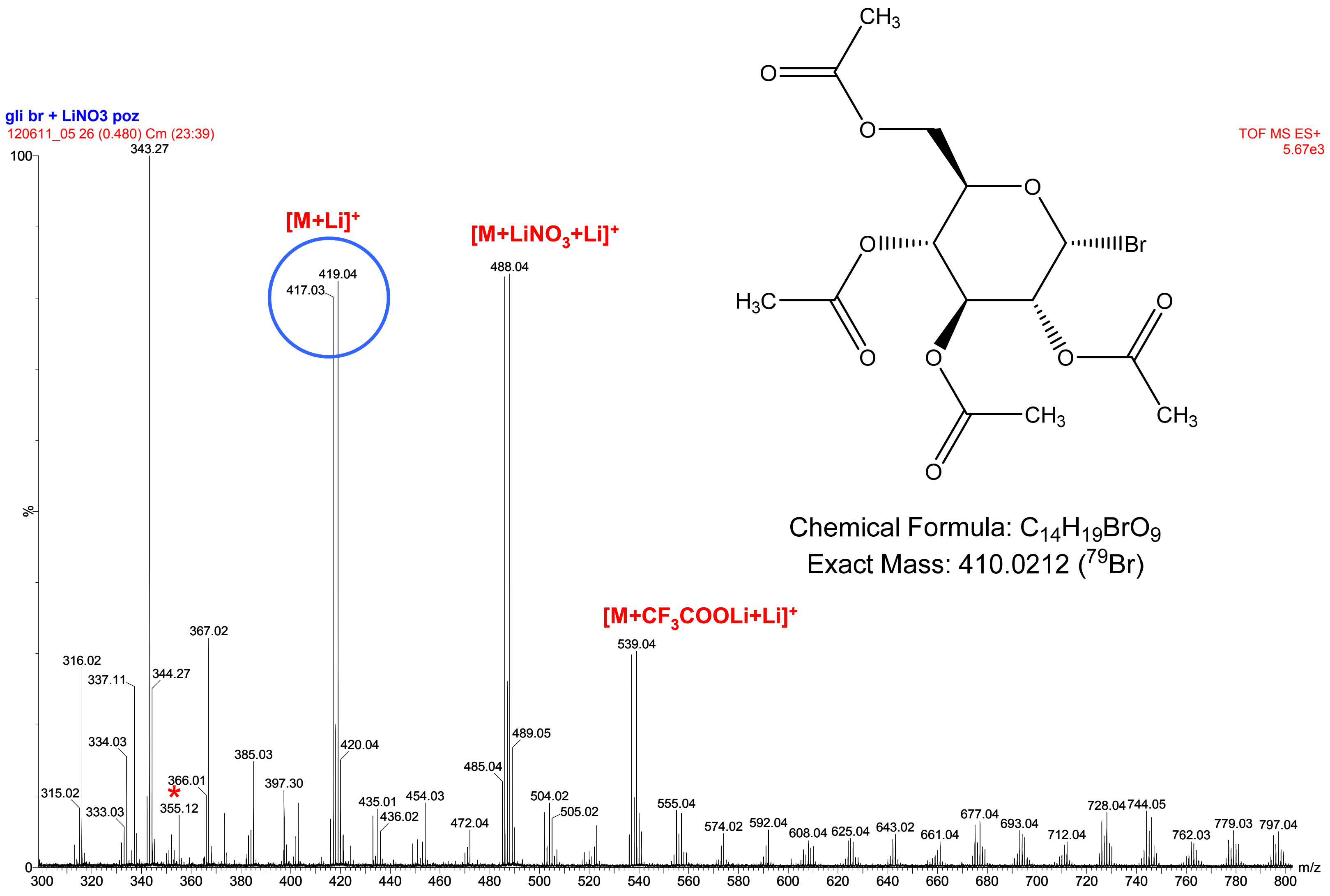

Spectrum 7. Analyte **2** in acetonitrile with $NaNO_3$.

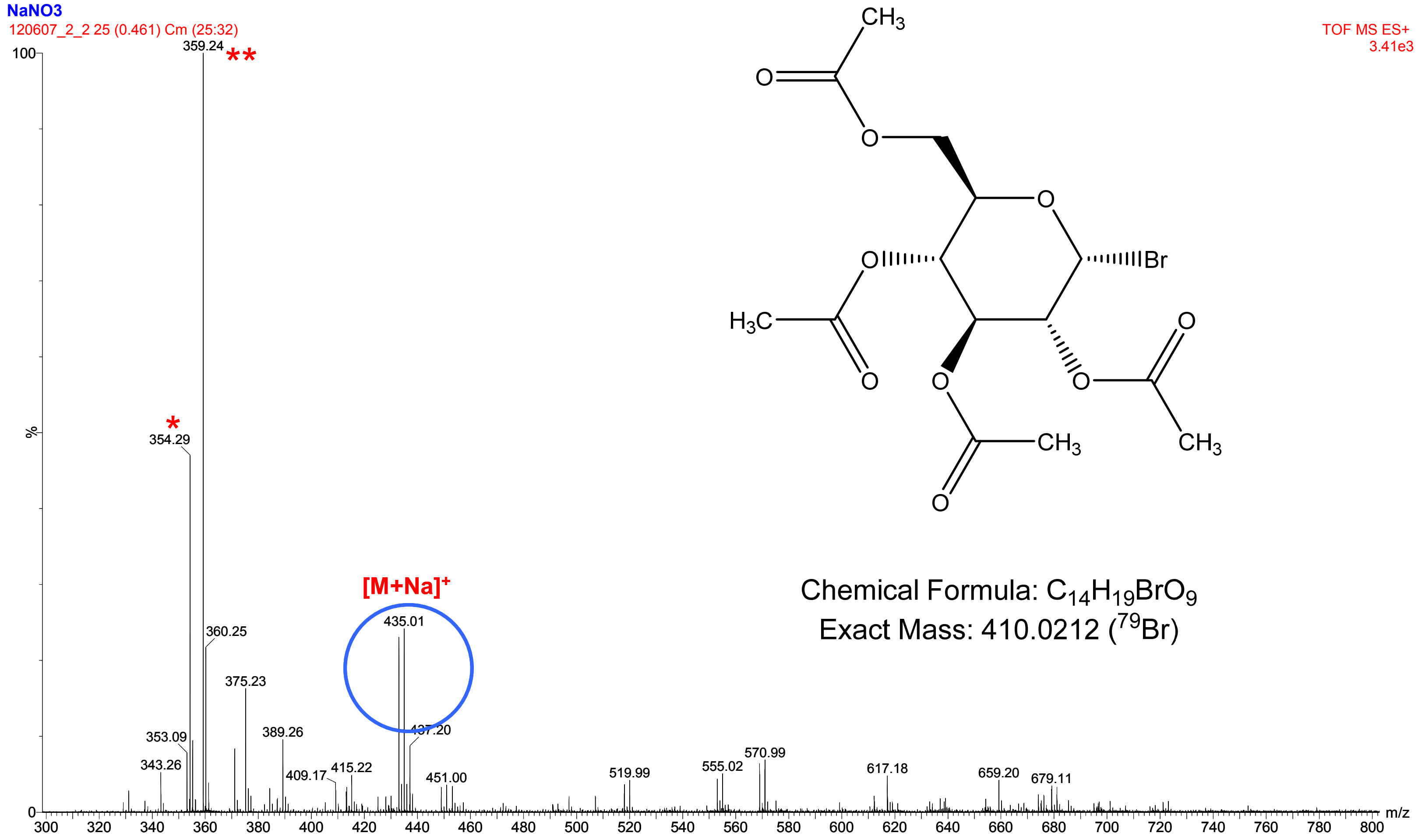

Spectrum 8. Analyte **2** in acetonitrile with KNO_3.

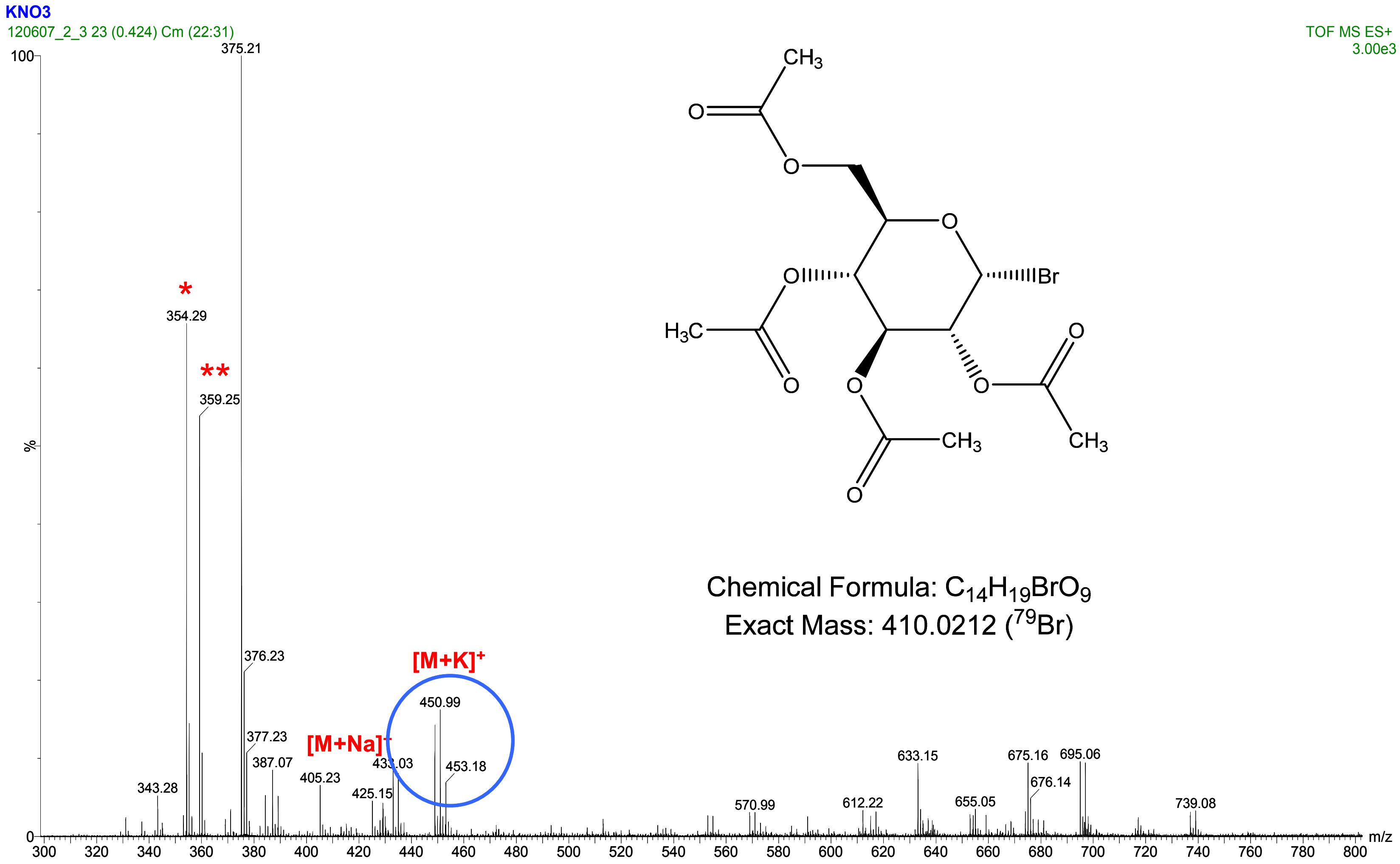

Spectrum 9. Analyte **2** in acetonitrile with $LiClO_4$.

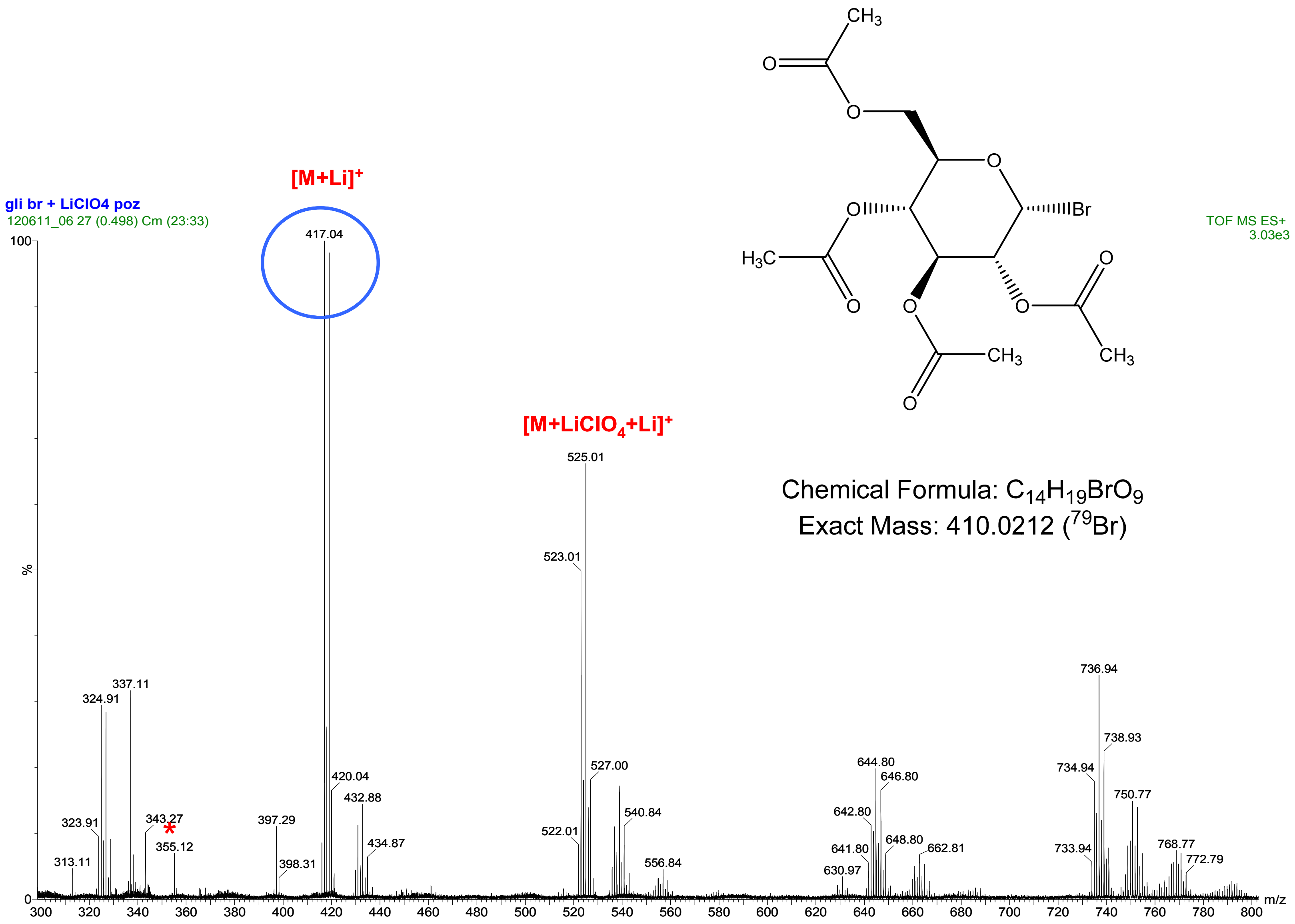

Spectrum 10. Analyte **2** in acetonitrile with $NaClO_4$.

Chemical Formula: $C_{14}H_{19}BrO_9$
Exact Mass: 410.0212 (^{79}Br)

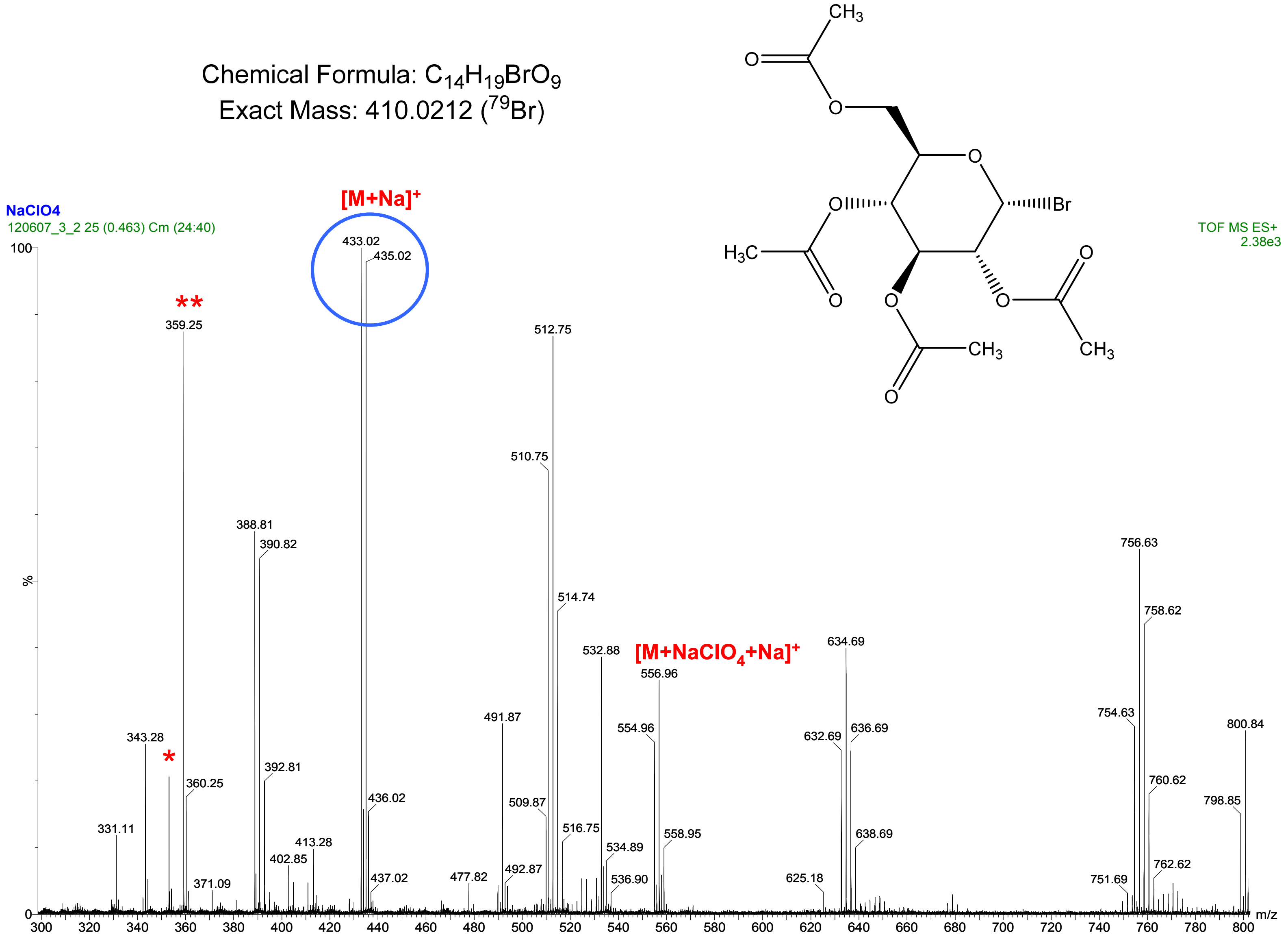

Spectrum 11. Analyte **2** in acetonitrile with $KClO_4$.

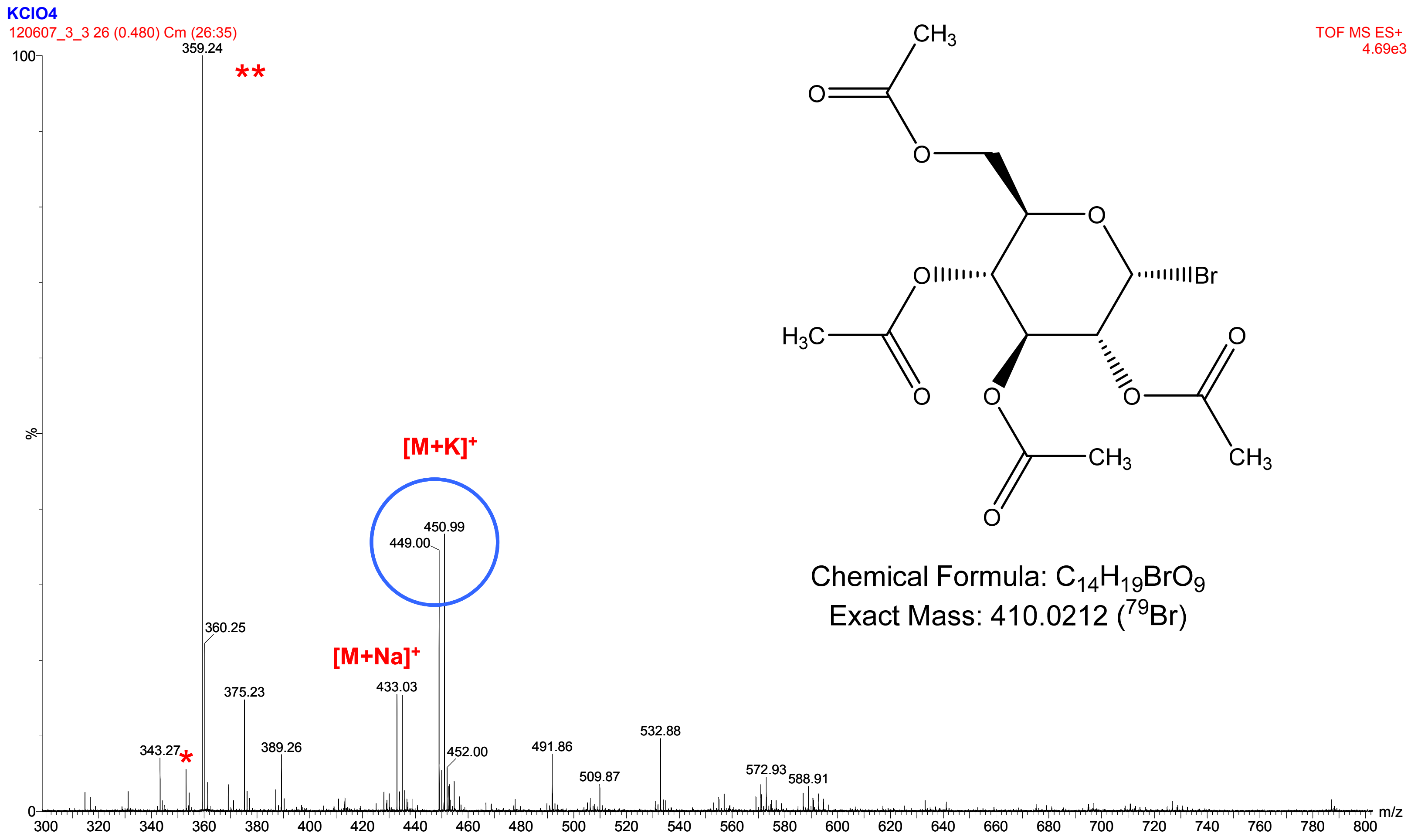

Spectrum 12. Analyte **2** in acetonitrile with NH_4Cl.

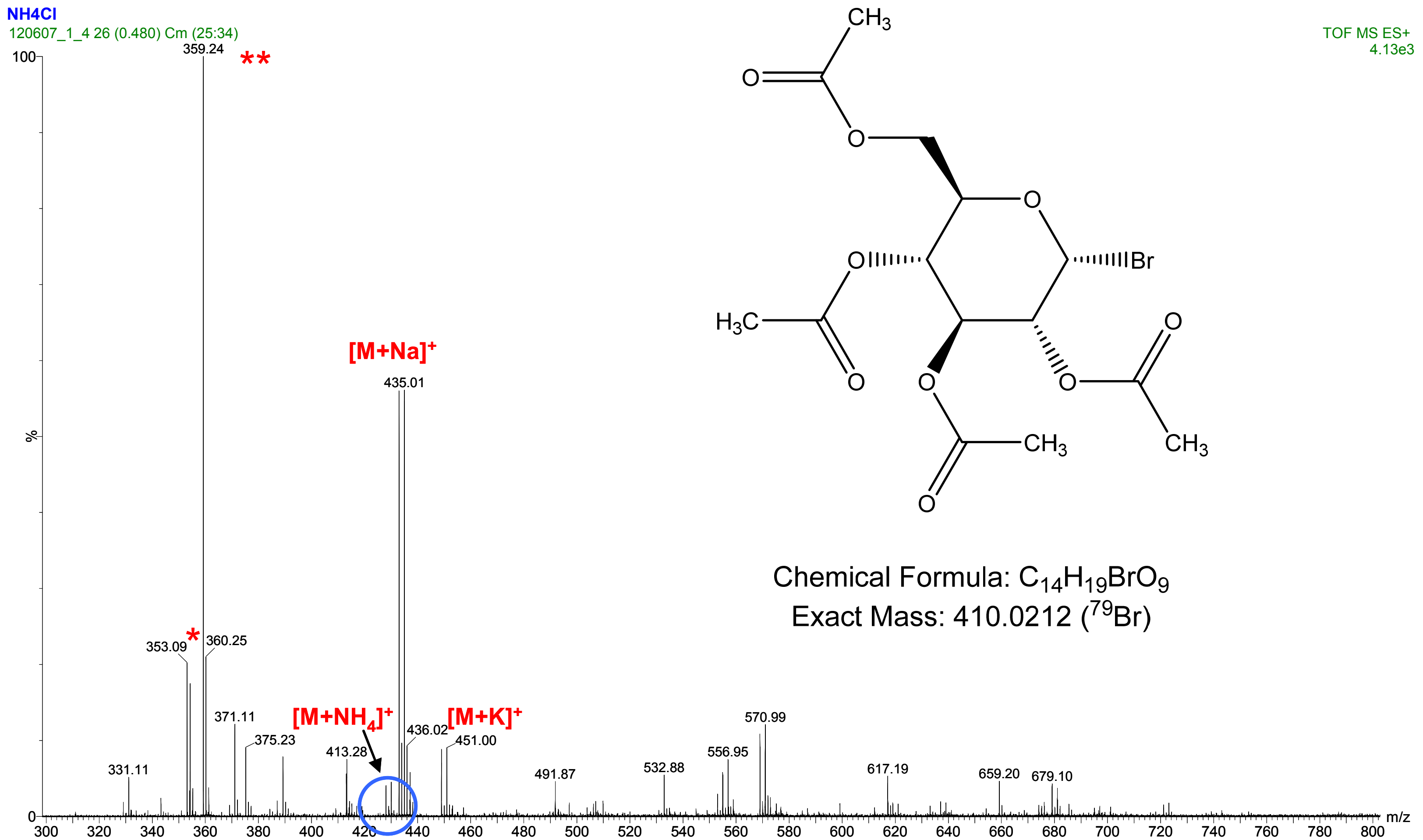

Spectrum 13. Analyte **2** in acetonitrile with NH_4NO_3.

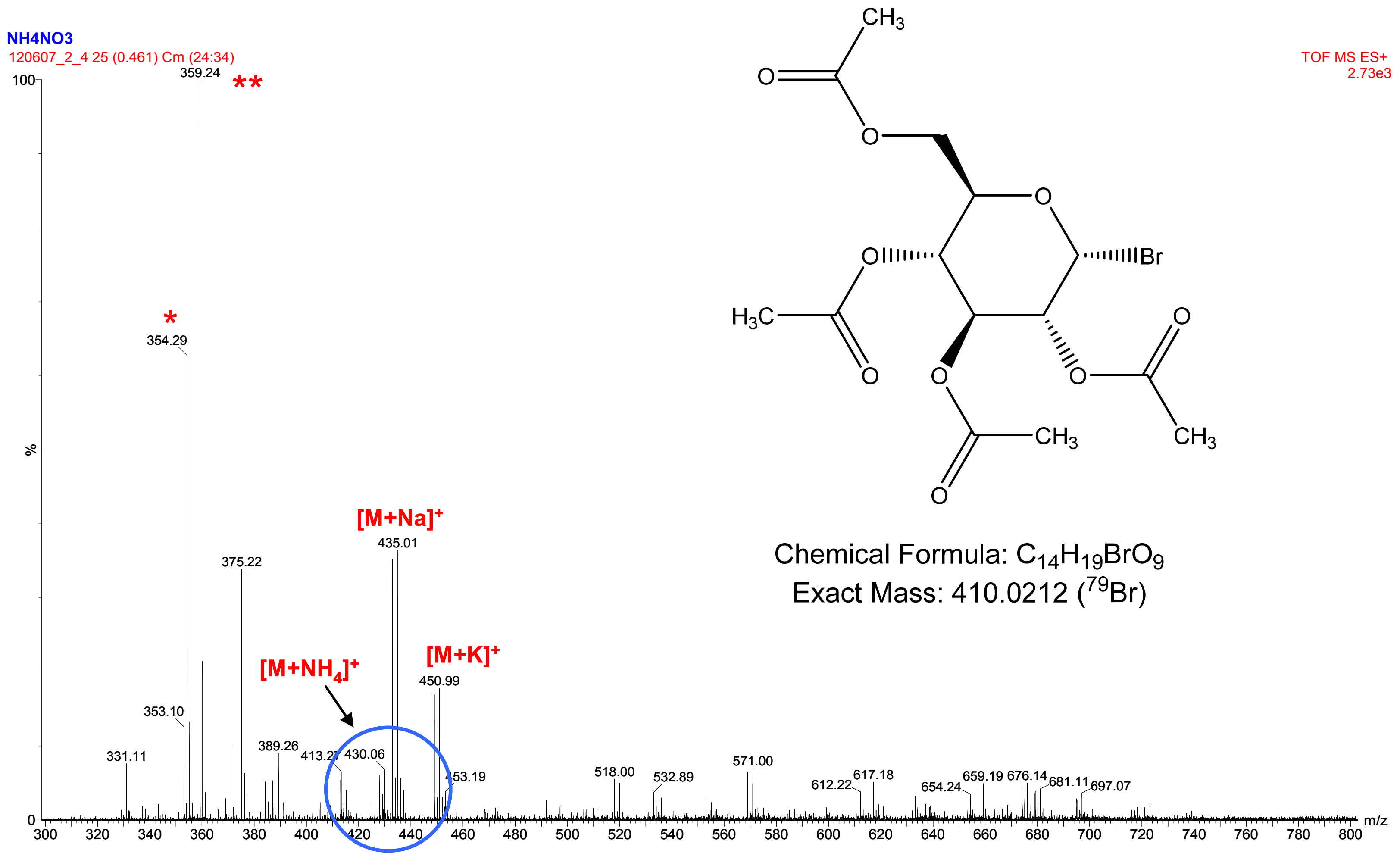

Spectrum 14. Analyte **2** in acetonitrile with NH_4OOCCH_3.

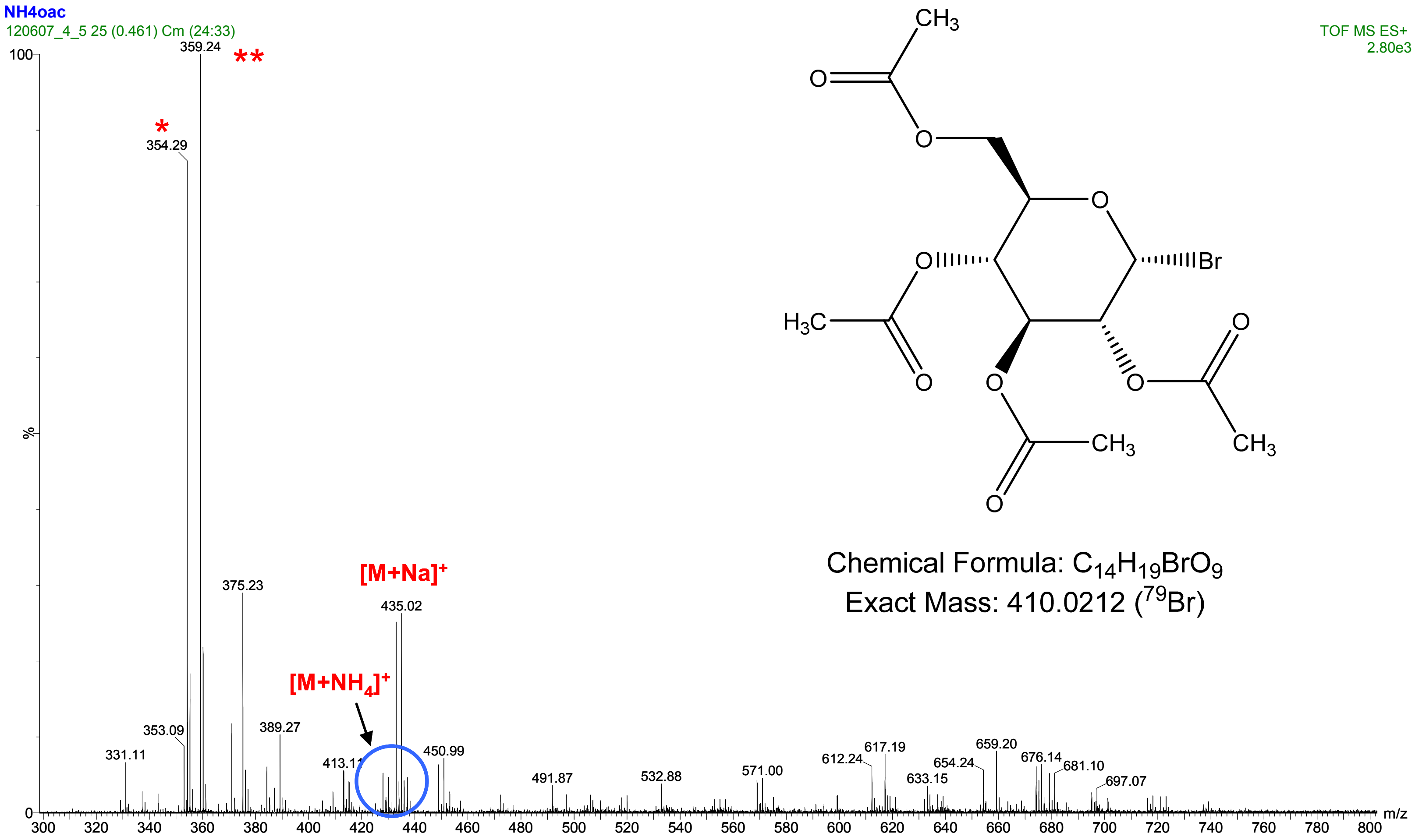

Spectrum 15. Analyte **2** in acetonitrile with NH_4OOCH.

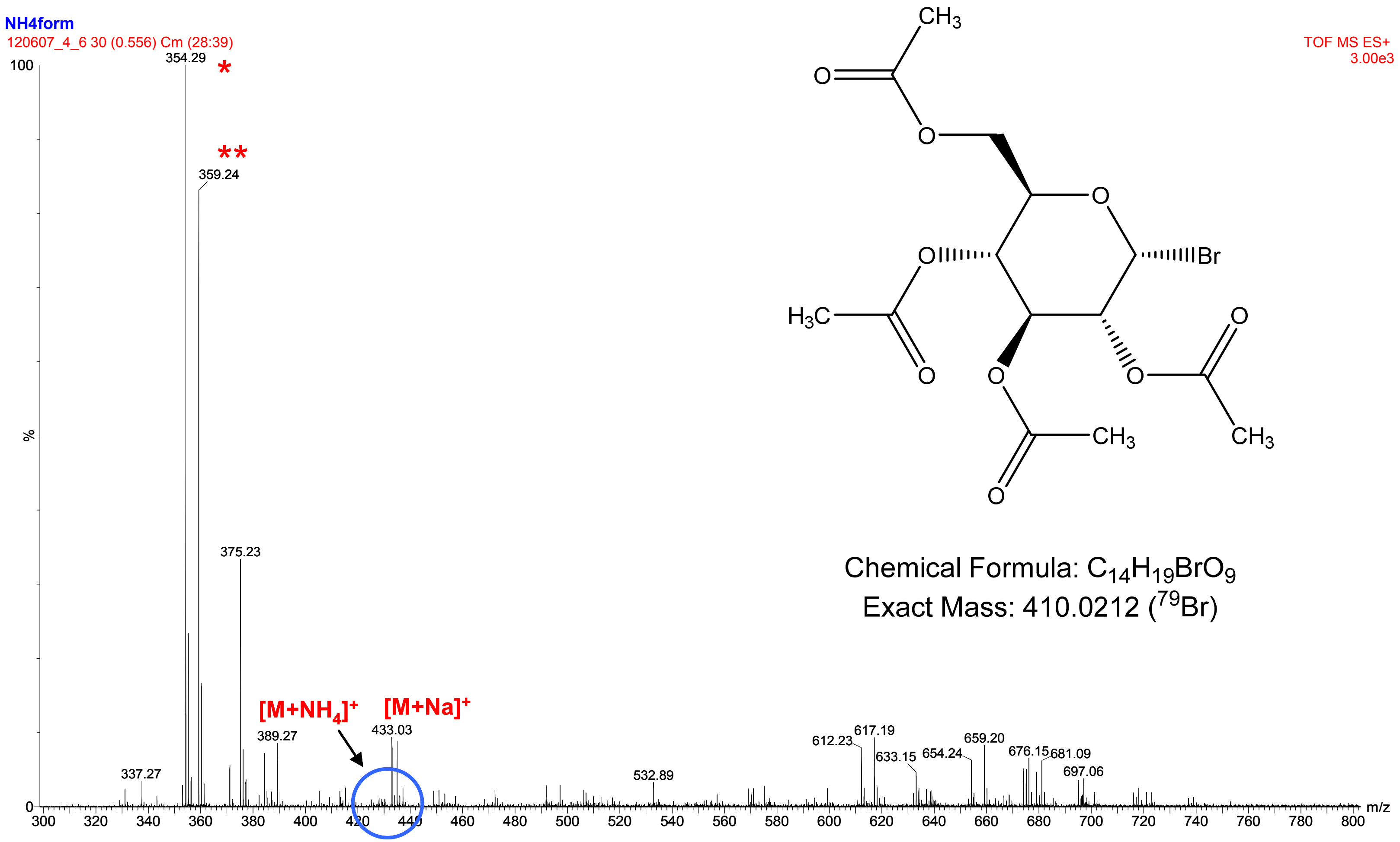

Chemical Formula: $C_{14}H_{19}BrO_9$
Exact Mass: 410.0212 (^{79}Br)

Spectrum 16. Analyte **2** in acetonitrile with NH_4HCO_3.

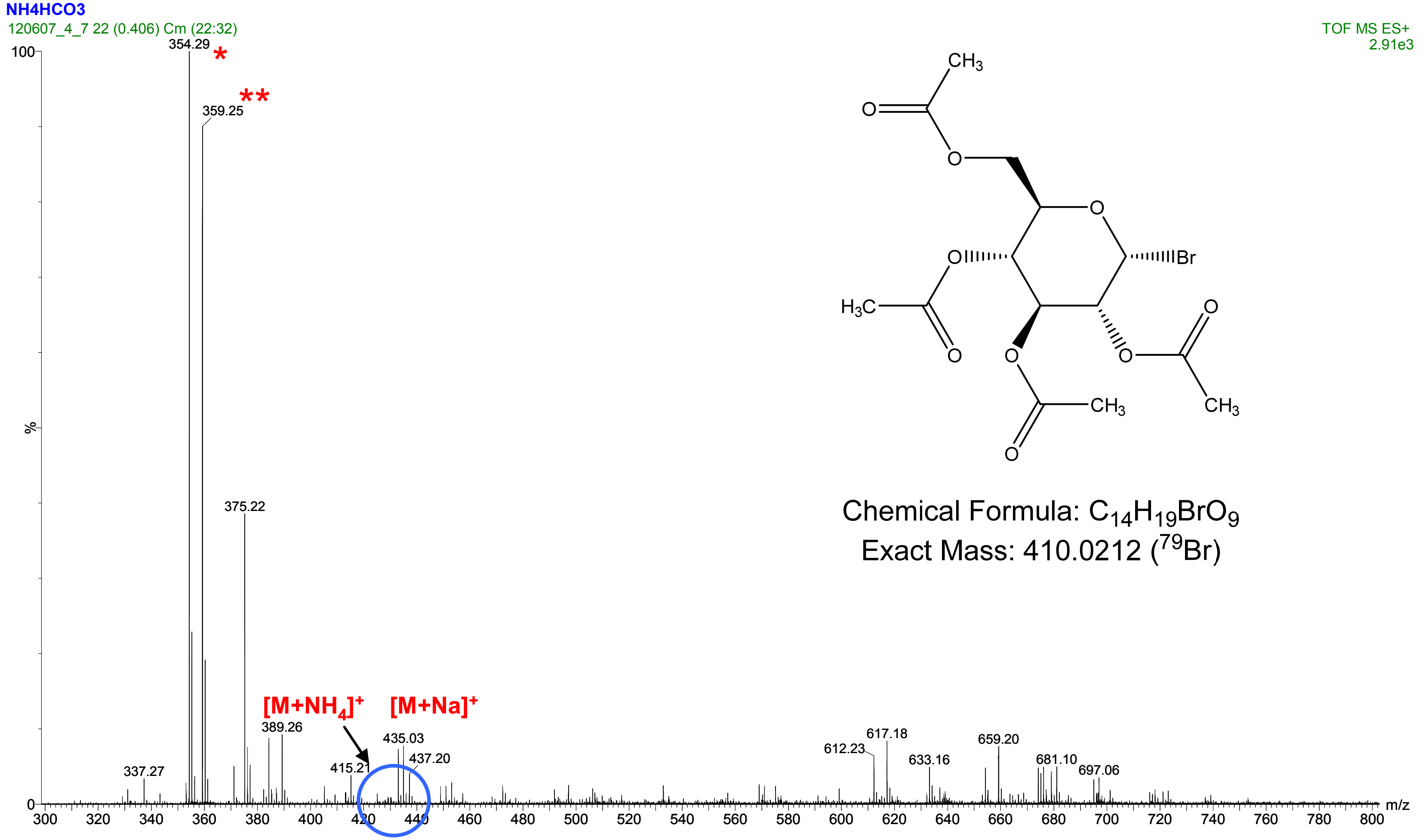

Spectrum 17. Analyte **2** in acetonitrile with LiBr.

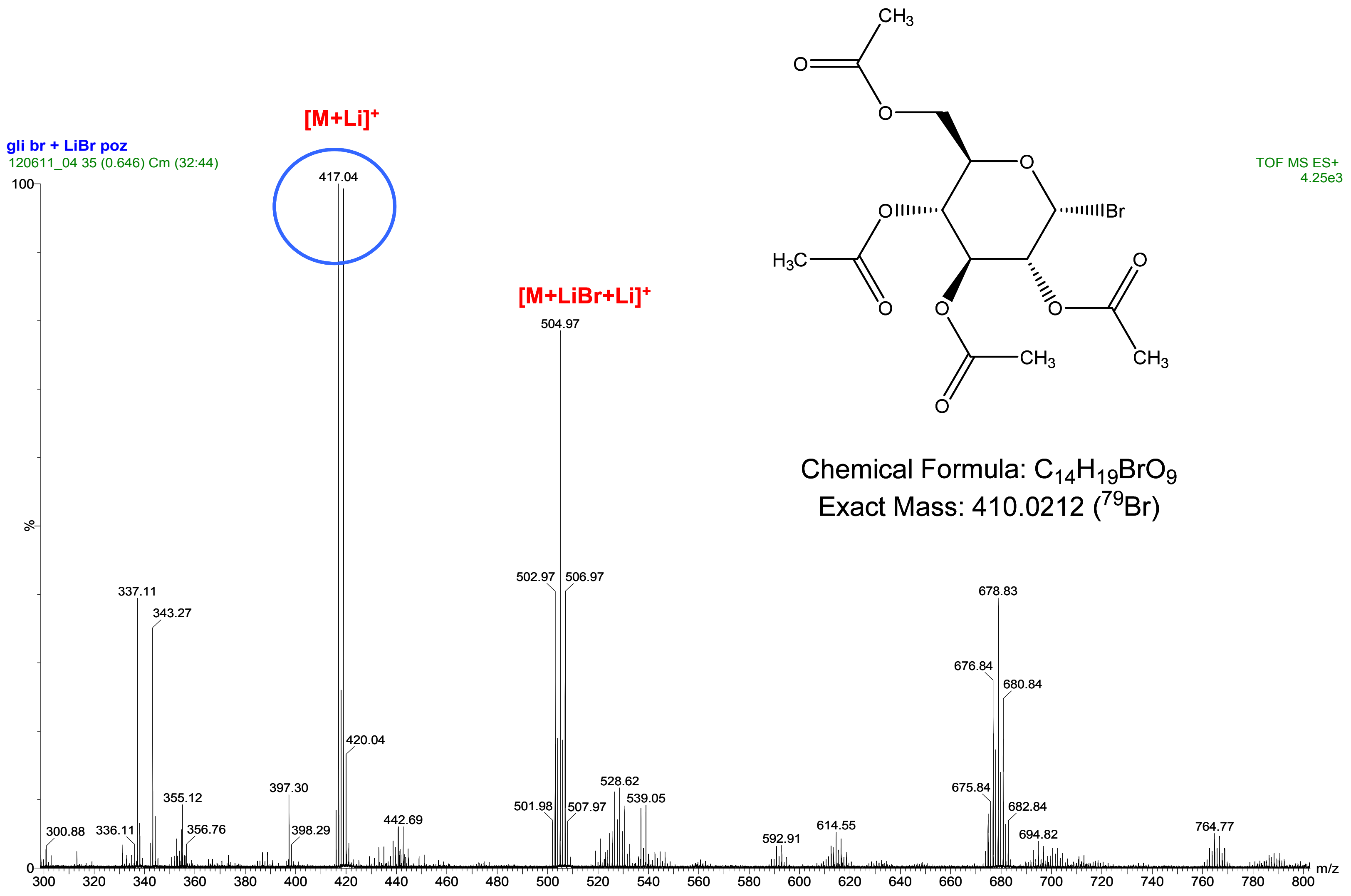

Spectrum 18. Analyte **2** in acetonitrile with LiI.

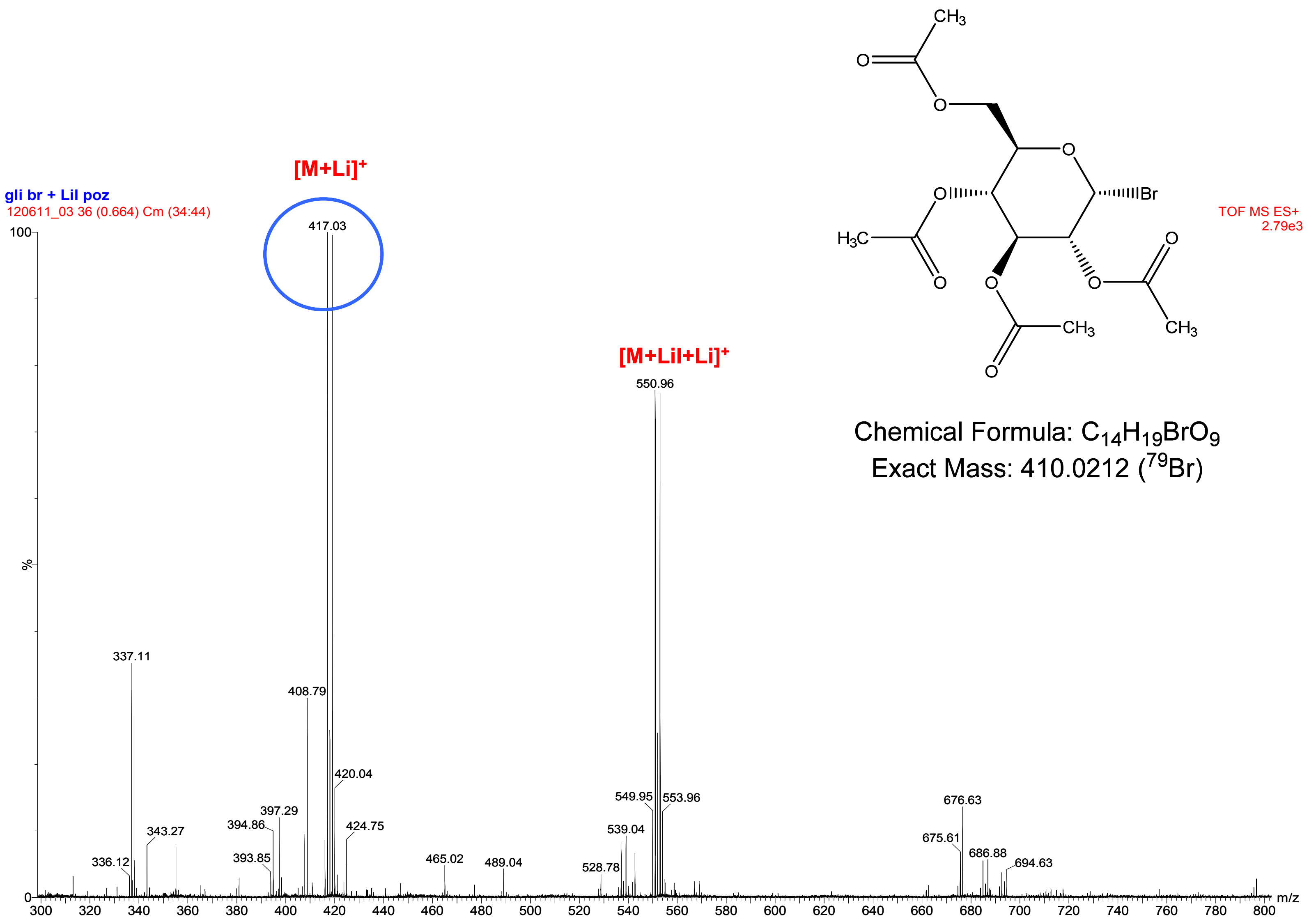

Spectrum 19. Analyte **2** in acetonitrile with LiF (negative ion mode).

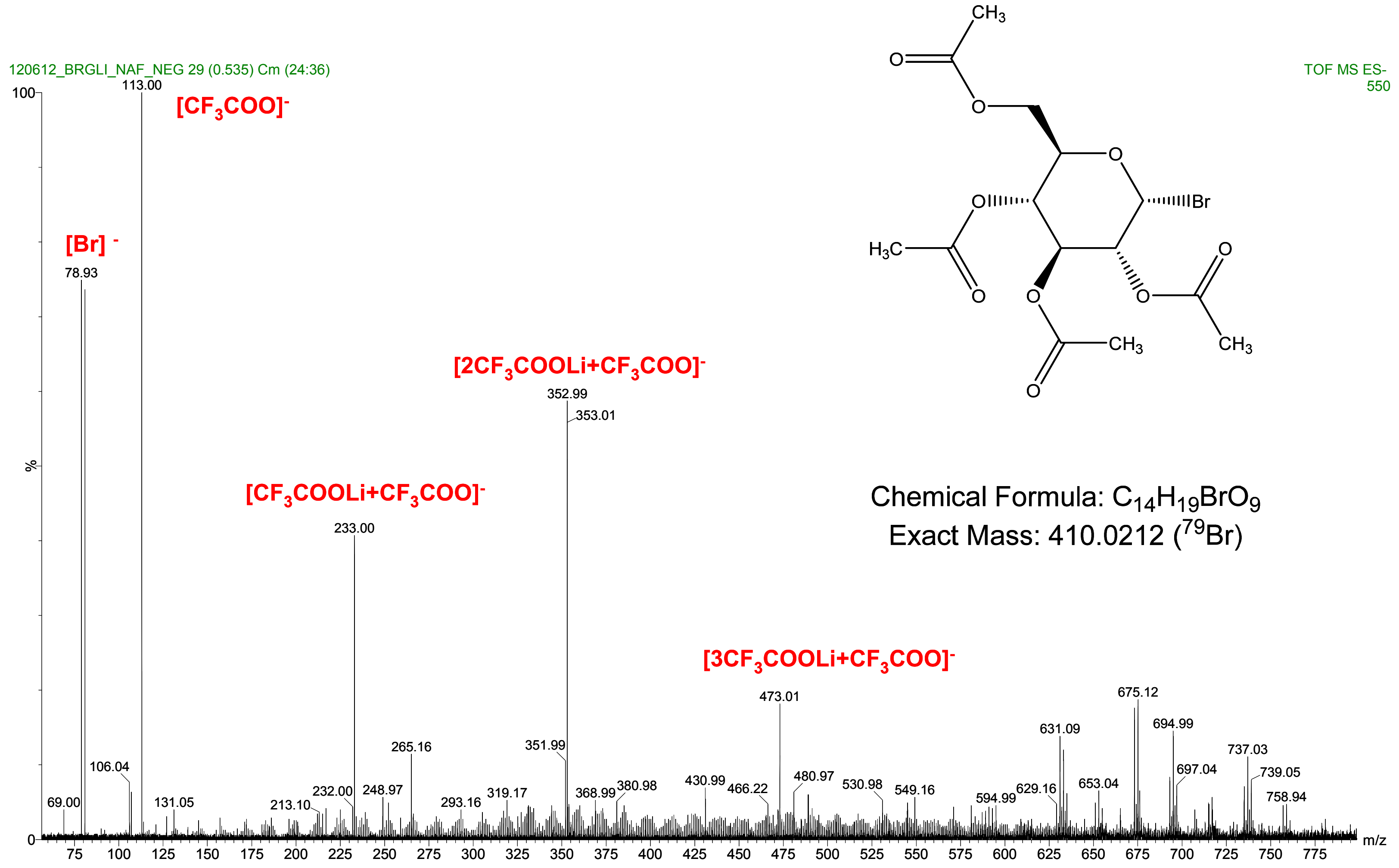

Spectrum 20. Analyte **2** in acetonitrile with LiCl (negative ion mode).

Chemical Formula: $C_{14}H_{19}BrO_9$
Exact Mass: 410.0212 (^{79}Br)

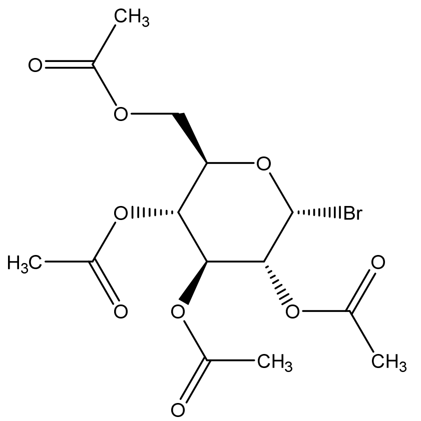

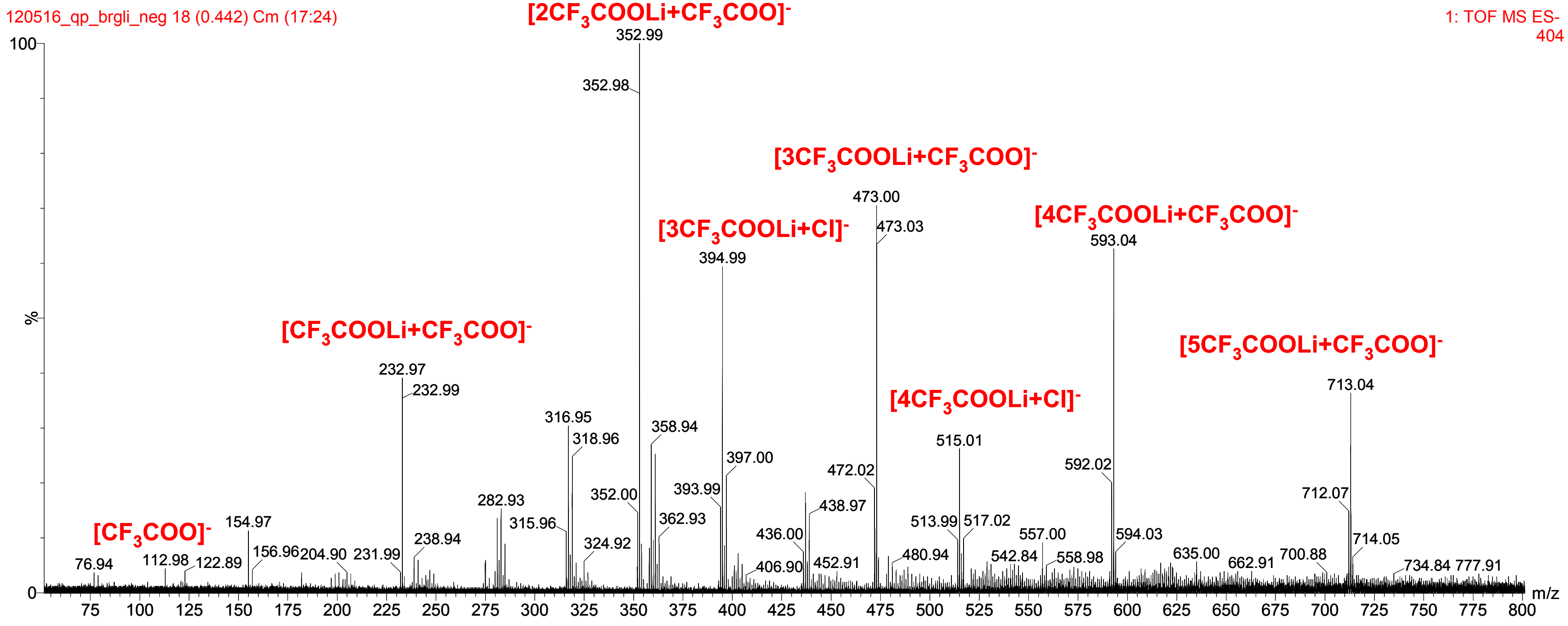

Spectrum 21. Analyte **2** in acetonitrile with LiBr (negative ion mode).

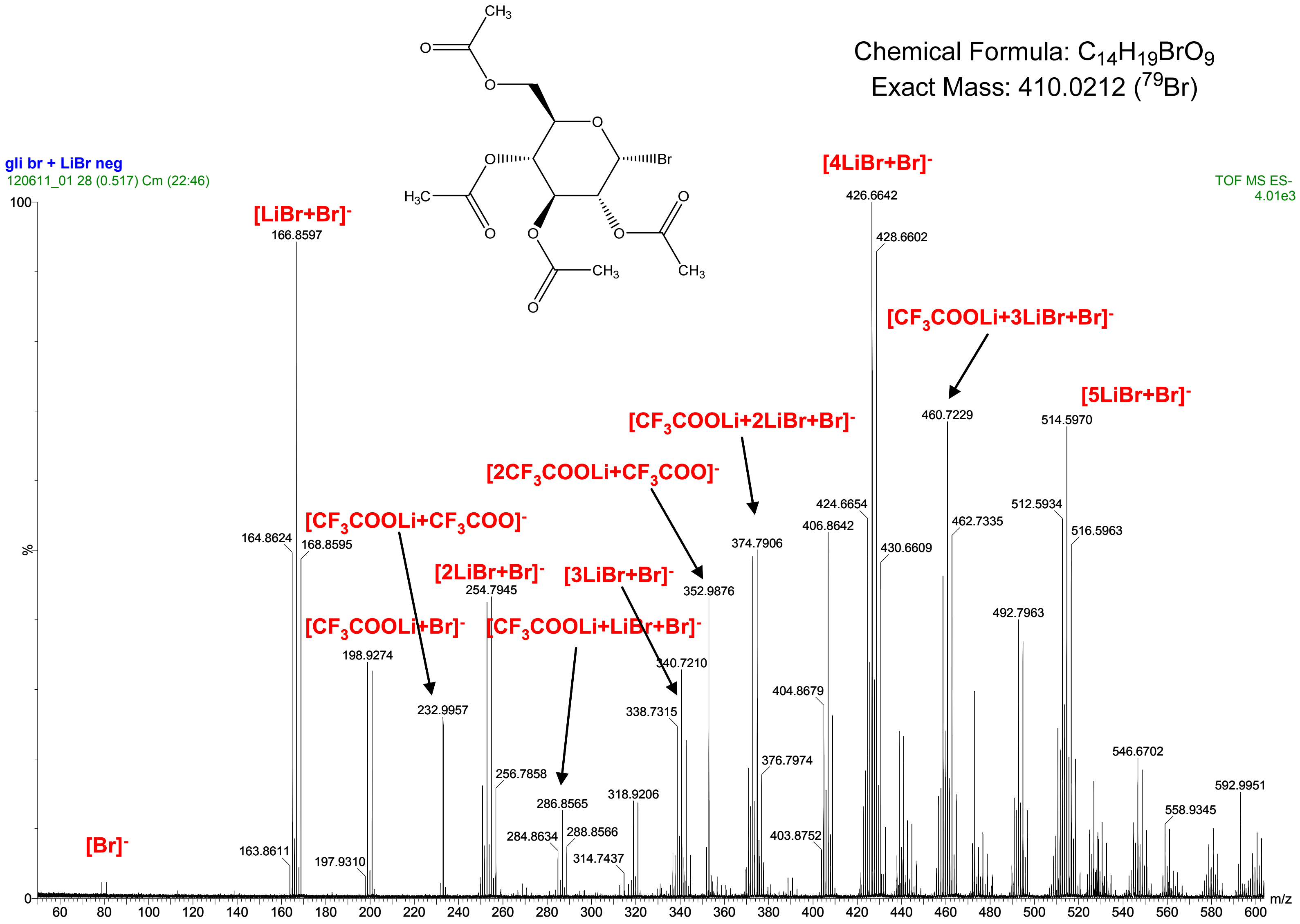

Spectrum 22. Analyte **2** in acetonitrile with LiBr (negative ion mode).

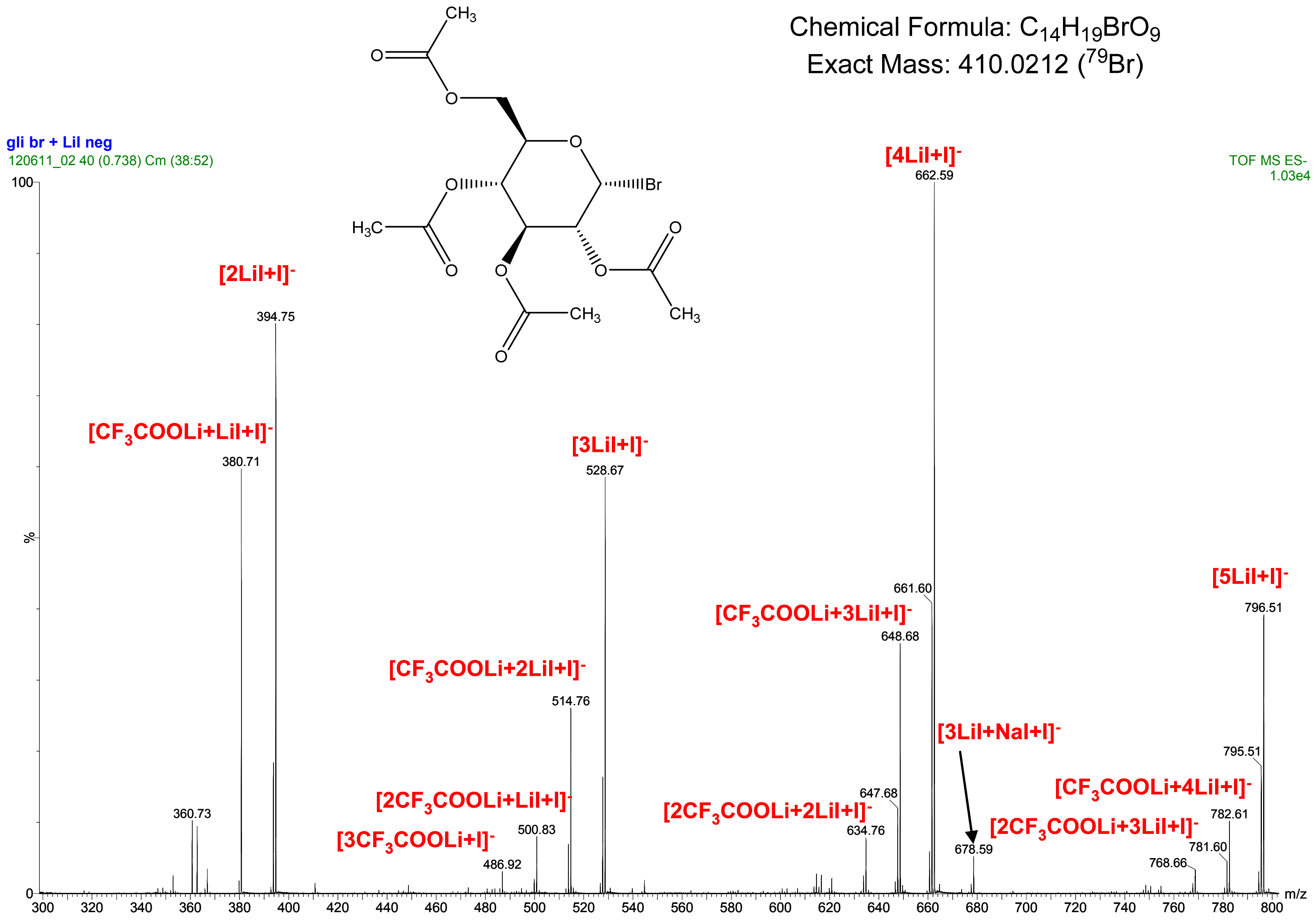

Spectrum 23. Analyte **2** in acetonitrile with NH_4OOCCH_3 (negative ion mode).

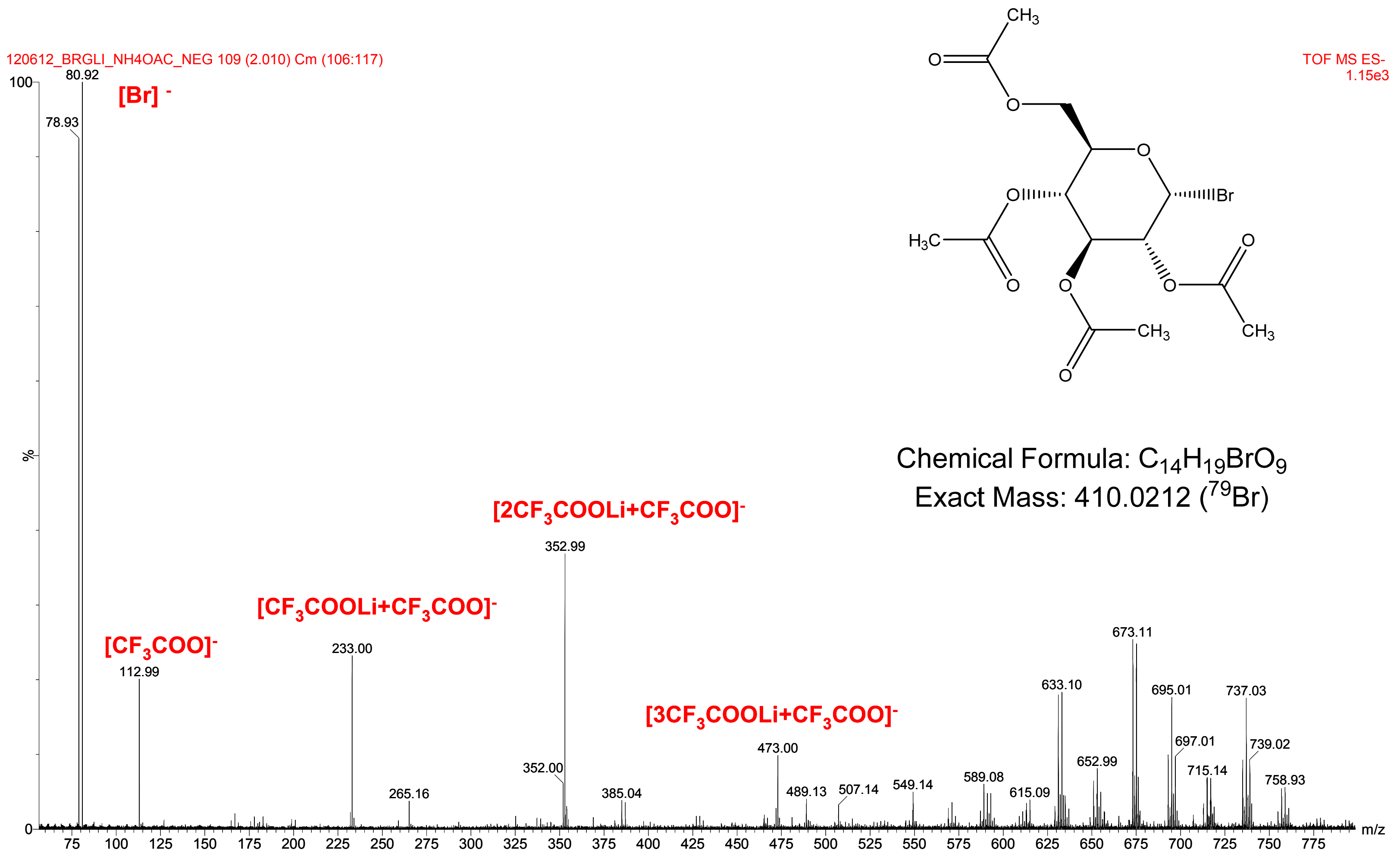

Spectrum 24. Accurate mass analysis of analyte **1** in acetonitrile with LiCl.

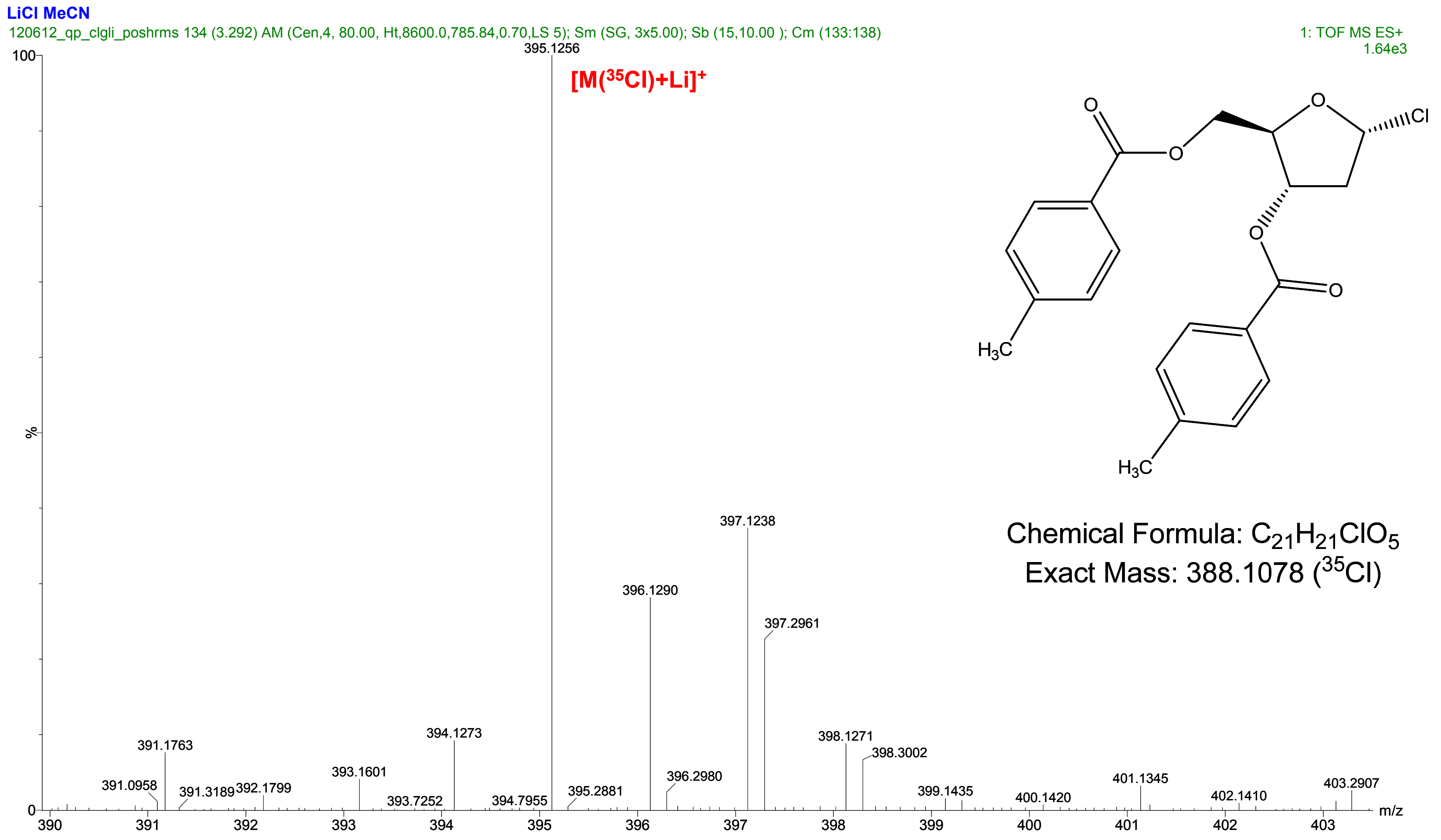

Table 25. Report of accurate mass analysis of analyte **1** in acetonitrile with LiCl.

Chemical Formula: $C_{21}H_{21}ClO_5$
Exact Mass: 388.1078 (^{35}Cl)

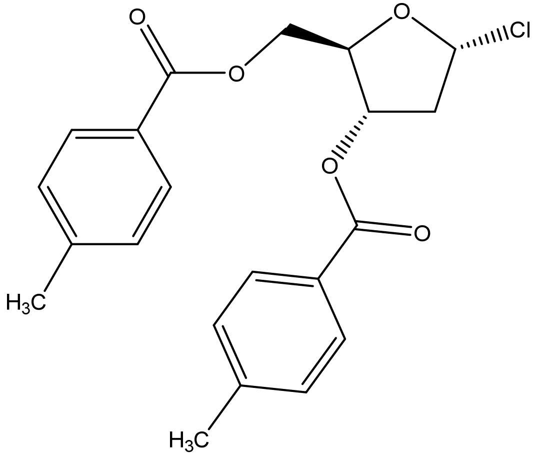

- Elemental Composition Report

- Single Mass Analysis
- Tolerance = 10.0 PPM / DBE: min = -1.5, max = 50.0
- Element prediction: Off
- Number of isotope peaks used for i-FIT = 3

- Monoisotopic Mass, Even Electron Ions
- 65 formula(e) evaluated with 1 results within limits (all results (up to 1000) for each mass)
- Elements Used:
- C: 0-500 H: 0-1000 7Li: 1-1 O: 0-20 35Cl: 1-1

Minimum:					-1.5			
Maximum:			5.0	10.0	50.0			
Mass (Norm)		Calc. Mass Formula		mDa	PPM	DBE	i-FIT	i-FIT
395.1256		395.1238 C21 H21 7Li O5 35Cl		1.8	4.6	10.5	97.5	0.0

Spectrum 26. CID spectrum of peaks 537/539 of analyte **2** in acetonitrile-LiCl at low collision energy.

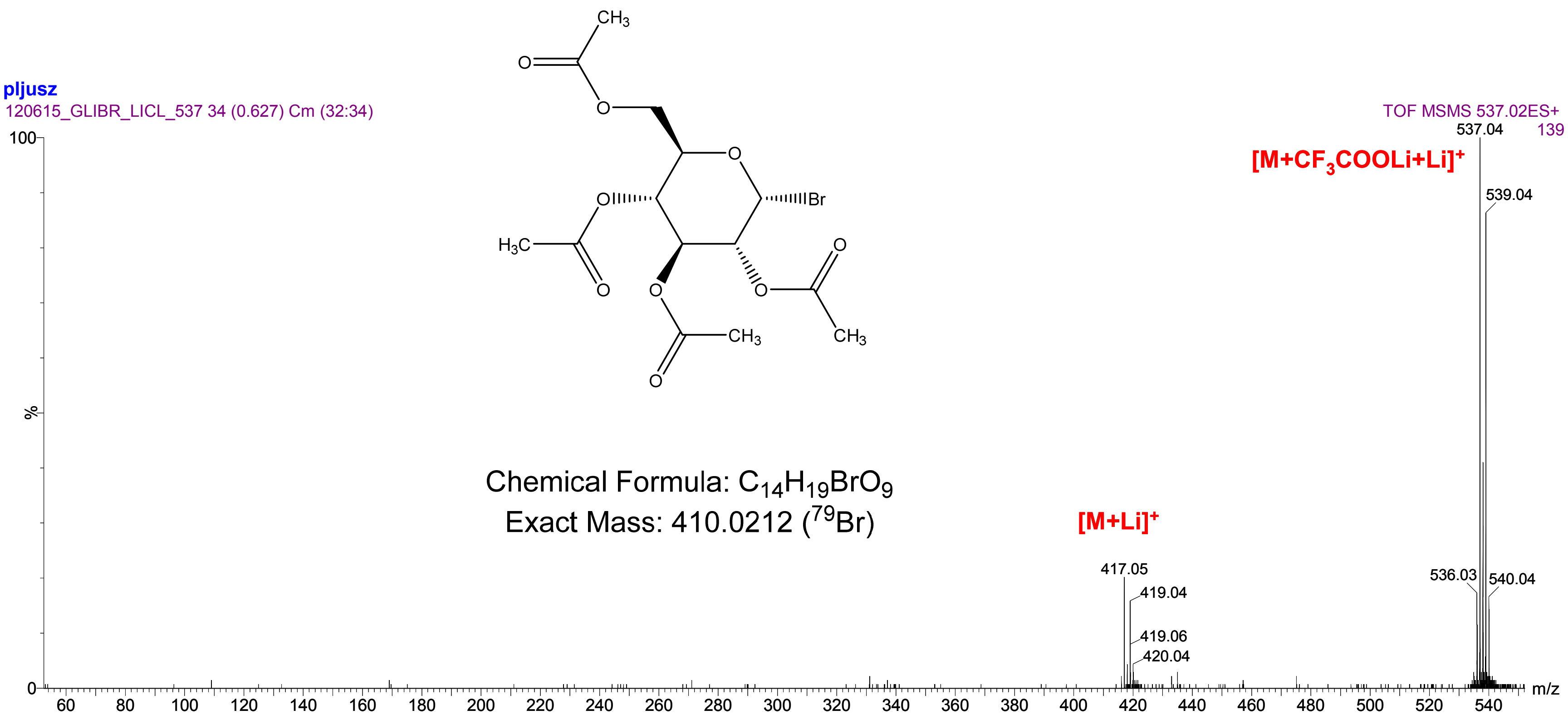

Spectrum 27. CID spectrum of peaks 417/419 and 537/539 of analyte **2** in acetonitrile-LiCl at high collision energy.

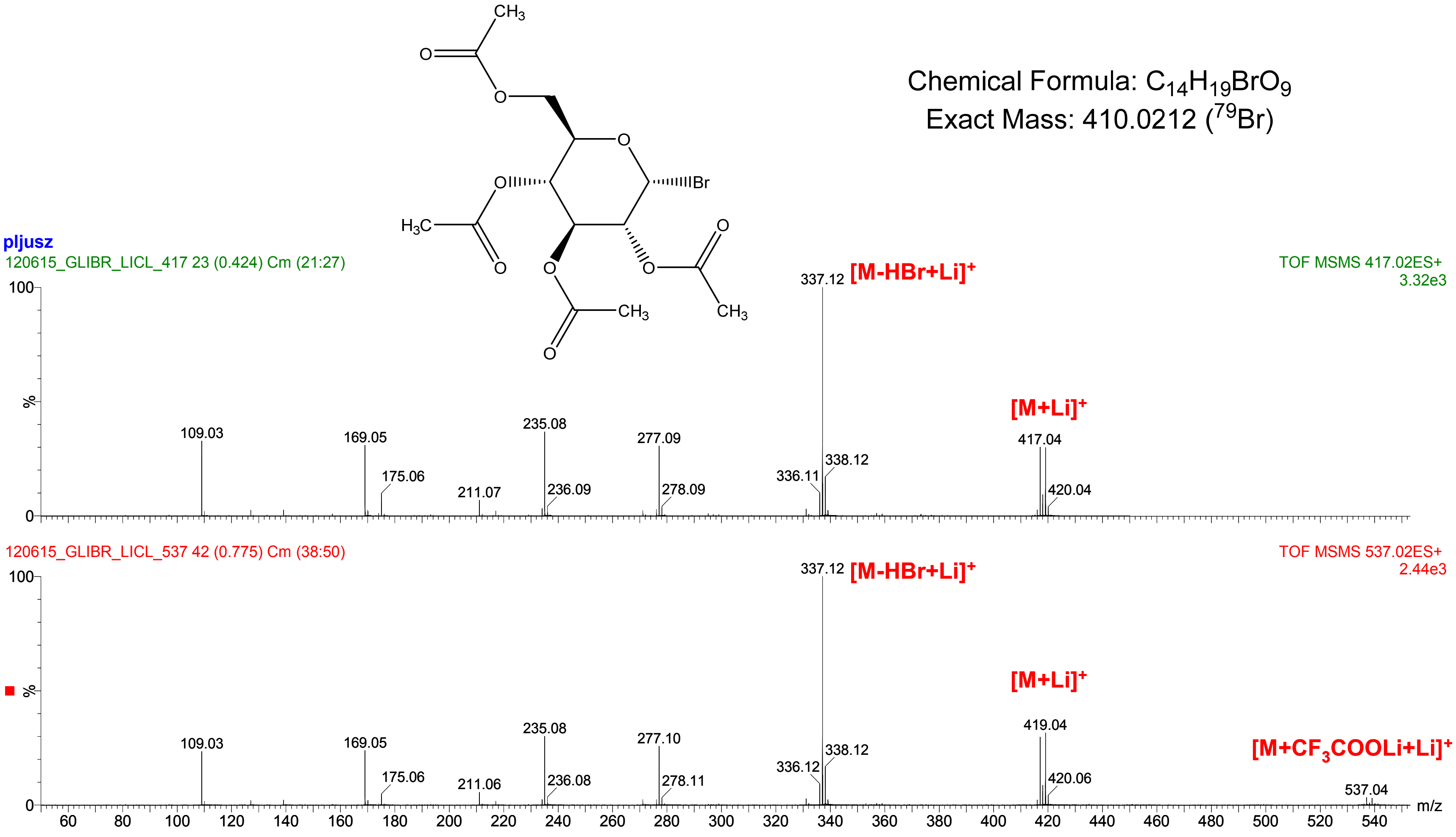

Supplement: Supplementary file 1 [file molecules-17-08351-s001.pdf]
